# Spatial patterns of CTCF sites define the anatomy of TADs and their boundaries

**DOI:** 10.1186/s13059-020-02108-x

**Published:** 2020-08-12

**Authors:** Luca Nanni, Stefano Ceri, Colin Logie

**Affiliations:** 1 Department of Electronics, Information and Bioengineering (DEIB), Politecnico di Milano, Piazza Leonardo da Vinci 32, 20133 Milan, Italy; 2 Department of Molecular Biology, Radboud Institute for Molecular Life Sciences, Faculty of Science, Radboud University, PO box 9101, 6500 HG Nijmegen, The Netherlands

**Keywords:** Chromatin architecture, TADs, TAD boundary conservation, CTCF binding site clusters, CTCF orientation patterns, Loop extrusion

## Abstract

**Background:**

Topologically associating domains (TADs) are genomic regions of self-interaction. Additionally, it is known that TAD boundaries are enriched in CTCF binding sites. In turn, CTCF sites are known to be asymmetric, whereby the convergent configuration of a pair of CTCF sites leads to the formation of a chromatin loop in vivo. However, to date, it has been unclear how to reconcile TAD structure with CTCF-based chromatin loops.

**Results:**

We approach this problem by analysing CTCF binding site strengths and classifying clusters of CTCF sites along the genome on the basis of their relative orientation. Analysis of CTCF site orientation classes as a function of their spatial distribution along the human genome reveals that convergent CTCF site clusters are depleted while divergent CTCF clusters are enriched in the 5- to 100-kb range. We then analyse the distribution of CTCF binding sites as a function of TAD boundary conservation across seven primary human blood cell types. This reveals divergent CTCF site enrichment at TAD boundaries. Furthermore, convergent arrays of CTCF sites separate the left and right sections of TADs that harbour internal CTCF sites, resulting in unequal TAD ‘halves’.

**Conclusions:**

The orientation-based CTCF binding site cluster classification that we present reconciles TAD boundaries and CTCF site clusters in a mechanistically elegant fashion. This model suggests that the emergent structure of nuclear chromatin in the form of TADs relies on the obligate alternation of divergent and convergent CTCF site clusters that occur at different length scales along the genome.

**Graphical abstract:**

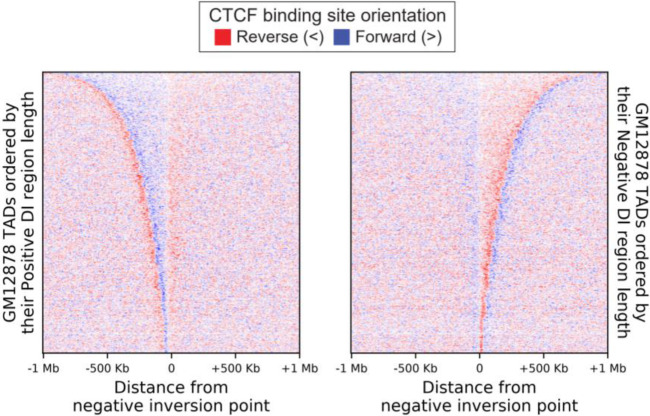

## Background

The 24 human chromosomes harbour slightly less than 20,000 protein-coding genes, of which more than 3500 exceed 100 kb in length, equivalent to more than 30 μm of DNA, when stretched-out. The biophysical phenomenology underlying storage of centimetre-sized chromosomes into sub-micrometre volumes is a current knowledge frontier as it is still unclear how sub-micrometre chromatin compaction is maintained while tens of micrometres of DNA are reeled through the catalytic sites of RNA polymerase II in a regulated fashion, so as to achieve proper gene expression levels.

It is generally accepted that transcription factors govern gene expression by recognising specific DNA sequences, so-called transcription factor binding sites, on gene promoters and enhancers. A major open question is how enhancers that are sometimes located more than 100 kb from their target promoters stimulate one or a given set of gene promoters rather than any promoter they could come in contact with inside the nucleoplasm [1–4].

Spatial segregation is a physical property; therefore, it can be investigated by slicing-up nuclei [5] or by using a molecular biology toolbox based on proximity ligation of restriction enzyme-cleaved formaldehyde-fixed nuclear DNA [6–8]. Proximity ligation has revealed the existence of chromosomal topologically associating domains (TADs) of 10^5^ to 10^6^ bp in size [9]. TADs are flanked by boundaries that are discovered as loci where there is a sharp break from preferential left-ward interactions to preferential right-ward interactions. In 2012, Dixon et al. thus modelled Hi-C data to detect 1723 human TAD boundaries [10, 11]. Similarly, the Blueprint consortium reported between 2800 and 3741 TADs in primary human blood cell types [12]. On the other hand, it was shown that convergent CTCF sites are located at the synapses between long-range interacting DNA regions, explaining multiple features of Hi-C data sets [13, 14]. Hence, the current view is that large chromatin loops are constrained at their basis by Cohesin rings [15–20] that accumulate at pairs of convergent CTCF sites [10, 13, 14, 21–31].

There are reports of more than 200,000 accessible CTCF motif instances in the human genome [32] and ENCODE reported more than 60,000 ChIP seq peaks for CTCF in a large number of human cell types [33]. Not all these CTCF sites necessarily form loops if we assume that convergent CTCF sites are brought together by a (semi)processive extrusion-like process that will start by folding a chromatin strand on itself followed by reeling-in DNA to extend the nascent loop [14, 15, 18, 34]. Indeed, Condensin and Cohesin extrusion reactions have recently been reconstituted in vitro, demonstrating ATP-dependent loop extrusion activity that can exert force on distal DNA tethers [35–37]. Additionally, it has recently being suggested that tandem arrays of CTCF binding sites regulate the spatial accessibility of sets of promoters in a balanced fashion [38].

Chromatin loops can be seen as the aggregated elements composing TADs, since in a single cell only one possible looping conformation can exist at any point in time, while TADs are the effect of the cumulative observations of conformations from several cells.

To reconcile TADs and CTCF site-mediated chromatin loops, we devised a classification scheme for clusters of more than two adjacent CTCF sites which is based on their relative orientation. Theoretical considerations demonstrate that the two-symbol CTCF grammar made of left- and right-pointing CTCF sites has the characteristic that convergent and divergent CTCF sites must alternate strictly along the length of chromosomes. This mathematical property of the CTCF grammar led us to hypothesise that divergent and convergent CTCF sites occur at different spatial regimes and that this underlies the emerging dimensions of TADs and their boundaries. We therefore analysed the distributions of CTCF cluster sizes along the human genome. This revealed that, at length scales ranging from 5 kb to 100 kb, divergent CTCF site clusters are enriched, while convergent CTCF site clusters are depleted, suggesting that divergent CTCF sites code for TAD boundaries and that convergent CTCF sites code for the left and right TAD sections. We validate the relative CTCF site orientation-based grammar as a function of CTCF site strength and TAD boundary strength. Finally, we investigate the CTCF grammar as a function of gene orientation and transcription intensity and find that, although more than one third of human gene promoters do harbour a CTCF site within 2 kilobase of the transcription start site, promoter CTCF sites are not stronger than non-promoter CTCF sites. We also find that CTCF site orientation is not linked to the direction of transcription, even though CTCF sites are most enriched in genes transcribed at high to intermediate intensities.

Altogether, our theoretical considerations and bioinformatic findings have fundamental implications for the discovery and engineering of CTCF-dependent chromatin domains and their boundaries.

## Results

### CTCF site stratification as a function of both ChIPseq signal and motif strength

In order to dissect the mechanistic roles of CCCTC-binding factor (CTCF) and the orientation of its DNA binding sites in human TAD structure, we started with a collection of 61,079 human CTCF sites reported by Rao et al. [13] (Additional file 2: Table S1). We analysed the conservation of the aforementioned binding sites using 33 ENCODE CTCF ChIPseq experiments [33] that encompass primary human tissue and cell lines (Additional file 3: Table S2). Globally, more than 16,000 CTCF sites are conserved in the 33 CTCF ChIPseq samples and over 40,000 are conserved in at least 20 samples (Additional file 1: Fig. S1A).

**Fig. 1:**
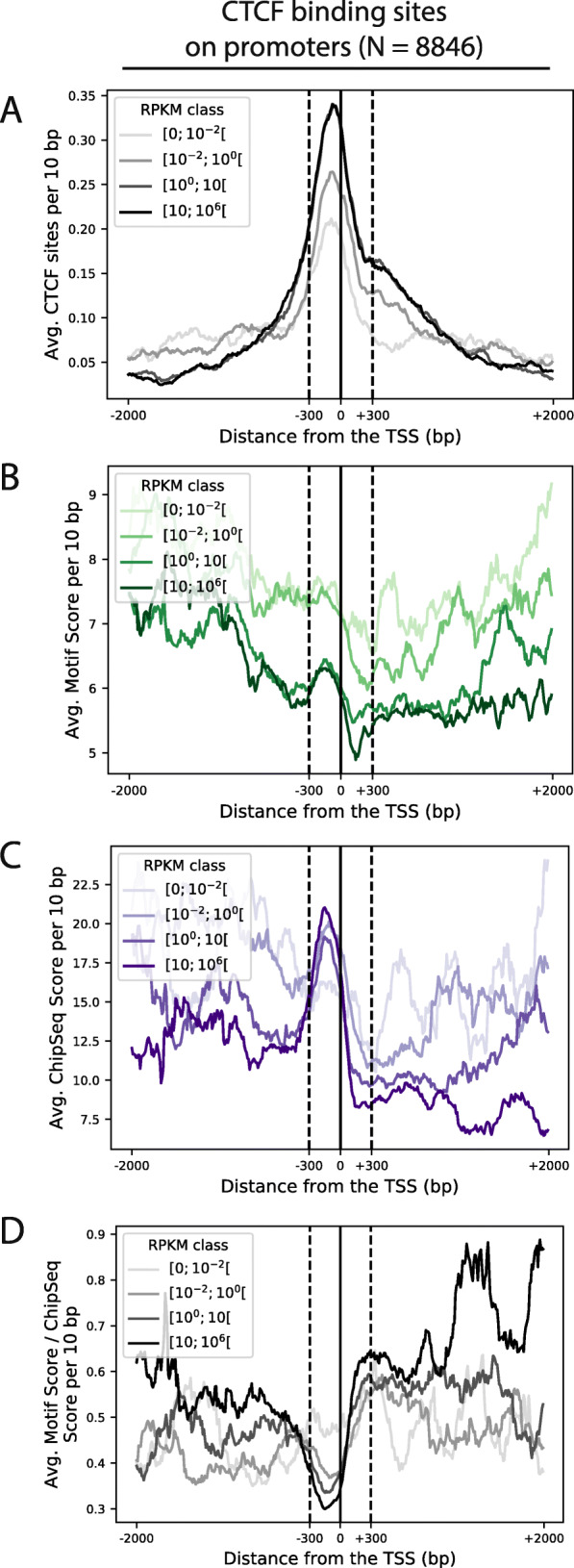
CTCF binding site enrichment at gene promoters. Density of CTCF binding sites and their features around 7747 gene promoters bearing at least one of 8846 CTCF binding sites computed as the averages in 10-bp bins. All panels are centred on the gene transcription start sites (Ensembl v72). The window starts on the left (− 2 kb) and ends in the gene body on the right (+ 2 kb). Genes are stratified in four equal subsets based on their expression values (Read Per Kilobase per Million reads, RPKM) in macrophages [4]; the shading of each line reflects the expression level. We first show **a** the density of CTCF binding sites, then **b** their average motif score computed by HOMER, then **c** their average ChipSeq score computed from the 33 ENCODE NarrowPeak tracks and finally **d** the ratios of average motif and ChipSeq scores for each bin

We then considered the strength of CTCF sites, which can be ranked by two measures: CTCF chromatin immunoprecipitation (ChIPseq) signal and CTCF binding site conformity to the consensus DNA sequence of the CTCF motif. We calculated both measures for each binding site of our collection (see the ‘Methods’ section) and found that they are generally concordant, although there appear to be many exceptions as indicated by a relatively low Pearson product-moment correlation coefficient of 0.43 (*R*^2^ = 0.18). A confounding factor underlying such variation may be the location of a CTCF site relative to gene features. In support of this hypothesis, CTCF site prevalence is highest in the vicinity of protein-coding gene promoters (Fig. 1a). We therefore investigated the potential for an altered relation between CTCF motif score (Fig. 1b) and CTCF ChIPseq signal (Fig. 1c) as a function of distance to transcription start sites (TSS) and gene transcription intensity [4] emanating from 7747 protein-coding gene promoters bearing at least one of 8846 CTCF sites located within 2 kb of the TSS. The lowest motif/ChIPseq score ratio (Fig. 1d) is achieved within a 300-bp window 5′ of the TSS, indicating efficient CTCF binding at these sites. This is presumably because promoter-associated ATP-dependent chromatin remodelling machines [39] facilitate DNA access for CTCF. The smoothed average ratio between motif strength and ChIPseq signal increases to double its value between the TSS and 150 bp 3′ downstream. This is maintained further downstream of the TSS when highly expressed (> 10 RPKM) genes are considered (Fig. 1d). We therefore speculate that engaged RNA polymerase II-associated complexes can compete with CTCF for DNA binding 3′ of the TSS.

Altogether, this suggests that CTCF binding can be modulated by gene promoter-associated chromatin remodelling factors and engaged RNA polymerases. Therefore, both ChIPseq signal and motif strength may have to be taken into account when computing CTCF site strengths. To this end, we associated an aggregate rank score to every Encode CTCF site by multiplying their ChIPseq signal and CTCF motif score ranks (see the ‘Methods’ section, Additional file 1: Fig. S1B-D, Additional file 2: Table S1).

The validity of this aggregated rank score in the context of genome conformation analysis was tested by chromatin loop simulation. We adopted a simplified model of loop extrusion [14] where the probability for the extrusion complex to stop at a CTCF motif is directly proportional to the selected CTCF feature, namely the motif, ChipSeq or rank aggregated score (see the ‘Methods’ section). We validated the predicted loops by comparing them with an independent set of long-range loops extracted by the HiCCUPS algorithm on the GM12878 cell line [13]. Since the aggregated rank score greatly outperformed the individual ChIPseq and motif rank scores when simulating loop extrusion, both in terms of recall and precision (Additional file 1: Fig. S1E-F), the notion that CTCF site strength is better described by combining motif score and ChIPseq signal is supported by our simulations.

With this validated CTCF site strength measure at hand, we could answer the following question: ‘Do the protein-coding gene promoter-bound CTCF sites dominate the CTCF landscape?’. Comparison of CTCF sites inside and outside of promoter regions shows that promoter CTCF sites are globally not stronger than the CTCF sites outside promoters, at the level of their Motif scores, ChIPseq signal and aggregate CTCF scores (Additional file 1: Fig. S2A, S2B). Furthermore, moderate to highly expressed promoters concentrate the majority of promoter CTCF sites, and these CTCF sites are usually not stronger than the genome-wide average (Additional file 1: Fig. S2C). We conclude that promoter-bound CTCF sites, which are presumably involved in long-range contacts with other gene features such as enhancers, do not dominate the global CTCF site landscape. Furthermore, in the GM12878 cell line, promoter CTCF site DNA methylation [40] does not appear be a confounder as most binding sites are not methylated (Additional file 1: Fig. S3).

Given this observation, in our downstream analyses, we decided to not filter CTCF sites based on gene features such as promoters.

### Classifying patterns of adjacent CTCF sites through their relative orientation

**Fig. 2:**
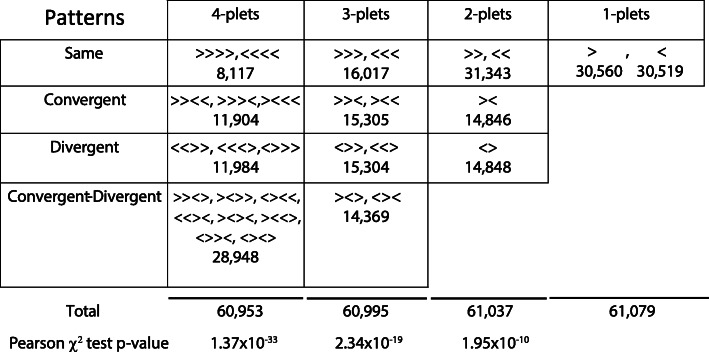
Classification of CTCF site clusters by relative orientation. CTCF mono-plet, di-plet, tri-plet and tetra-plet adjacent binding sites in all possible patterns of relative orientation. Patterns are divided into four classes: *Same* (all sites oriented in the same direction), *Convergent* (sites pointing towards each other), *Divergent* (sites pointing away from each other) and, for tri-plets and tetra-plets, the class *Convergent + Divergent*. The total number of patterns discovered from the complete set of CTCF binding sites in the human genome, independent of inter-CTCF site distance, is shown for each class. Note that the marginal sums of patterns along the columns are slightly different. This is because the number of *k-*plet patterns found in each chromosome arm (see the ‘Methods’ section) is equal to *M* − *K + 1*, where *M* is the total number of CTCF sites on that chromosome arm. Therefore, we have a discrepancy of 42 di-plets, 84 tri-plets and 126 tetra-plets relative to the mono-plets (see the ‘Methods’ section). *p* values are computed for di-, tri- and tetra-plets using the Pearson chi-square test. Effect sizes and significances were also computed by randomising the orientations of CTCF binding sites (see Additional file 1: Fig. S4)

Given that the bases of chromatin loops have been reported to involve pairs of convergent CTCF sites [13, 14], we hypothesised that classifying CTCF sites as a function of their orientation and that of their neighbours would yield novel mechanistic insight. We therefore took a genome-wide view of the spatial distribution of CTCF sites and the orientation of their motifs. The genome can be seen as a sequence of CTCF binding sites interrupted by spaces of variable length, which we term *inter-CTCF distance*. This sequence can be studied as a composition of its subsequences, which can overlap and can be designated to have a fixed number of sites. We can therefore assign a class to each subsequence based on the relative orientations of its binding sites. In the following, we analyse subsequences composed of one up to four CTCF sites (Fig. 2).

Formally, for individual CTCF sites (mono-plets), there are two orientation classes, namely forward/right-pointing (>) and reverse/left-pointing (<). For pairs of sites (di-plets), there are three classes; two patterns are oriented in the same direction (>>, <<), and there are also one convergent (><) and one divergent (<>) patterns. For sequences of three sites (tri-plets), there are two same-oriented (>>>, <<<), two convergent + same oriented (>><, ><<), two divergent + same (<>>, <<>) and two convergent + divergent (<><, ><>) patterns. This yields eight distinct orientation patterns divided in four orientation classes that we call ‘Same’, ‘Conv.’, ‘Div.’ and ‘Conv.+Div.’. For sequences of four sites (tetra-plets), there are 16 patterns; two Same, three Conv., three Div. and eight Conv.+Div. (Fig. 2). We limited our analysis up to tetra-plet patterns in order to prevent combinatorial explosion and also because longer sequences can be studied as a composition of their subsequences. In particular, the tri-plet classification system suffices for a complete description of all possible spatial patterns along the length of a chromosome.

A fundamental property of the distribution of CTCF site orientation patterns is that convergent (><) and divergent (<>) CTCF sites must have the same cardinality, as they change the direction of the system from right- to left-pointing and from left- to right-pointing, respectively, while this is not true for the Same and Conv.+Div. classes (see the ‘Methods’ section). For the collection of 61,079 human CTCF sites that we used here, there are 16,017 ‘Same’ CTCF sites and 14,369 ‘Conv.+Div.’ CTCF sites, indicating an evolutionary gain of same-oriented tri-plets (Pearson chi-square test *p* value = 2.34 × 10^−19^). By contrast, as asserted above, there are 15,305 Conv. (>><, ><<) and 15,304 Div. (<>>, <<>) CTCF tri-plet sites along the human chromosomes (Fig. 2, Additional file 2: Table S1). The unbalance between CTCF pattern classes increases with the complexity of the pattern from di-plets (*p* value = 1.95 × 10^−10^) to tetra-plets (*p* value = 1.37 × 10^−33^). To further validate our findings at the statistical level, we also calculated an empirical significance value by randomising the orientations of the single CTCF sites in the genome followed by counting the number of patterns in each class for each randomisation (Additional file 1: Fig. S4). This indicates that the unbalances shown in Fig. 2 are extremely significant (below machine resolution) for the di-, tri- and tetra-plet classes (see the ‘Methods’ section). We therefore propose that the mathematical property of obligate alternation of Conv. and Div. CTCF sites, together with a prevalence of Same tri-plets over Conv.+Div. tri-plets, underly the logic of chromosomal chromatin loop network organisation that gives rise to TAD-associated features such as TAD boundaries.

### Spatial distribution analysis of CTCF site clusters reveals robust orientation biases

**Fig. 3:**
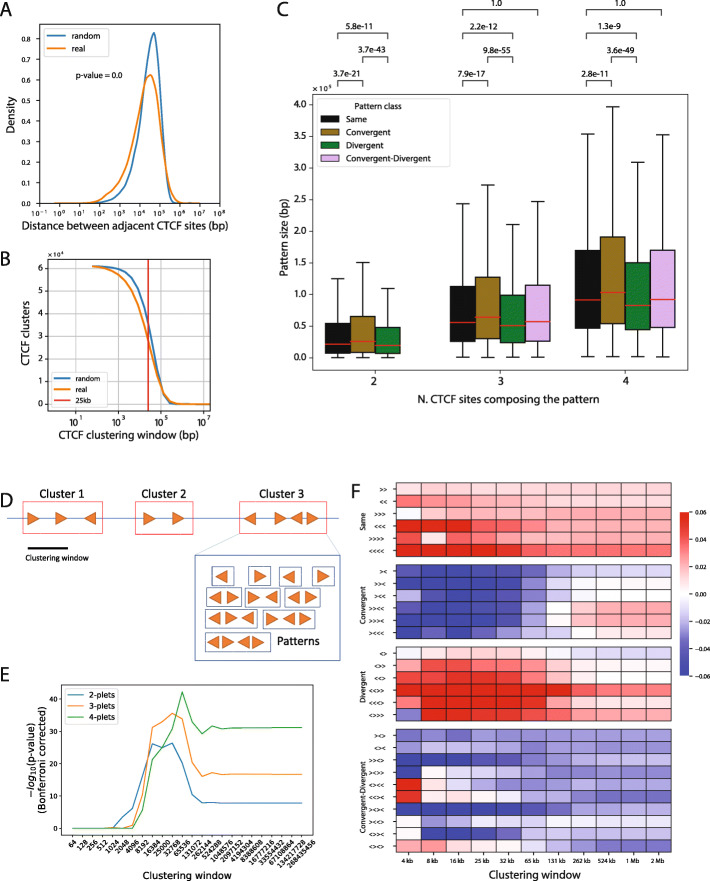
CTCF site cluster spatial distribution analysis reveals orientation biases in the human genome. Spatial distribution of CTCF binding sites and their motif orientation. **a** Distribution of distances between adjacent CTCF binding sites along the human genome (orange) and in spatially randomised CTCF binding sites (blue). The *p* value was computed at the hand of the Mann–Whitney *U* test for the difference between the two distributions. **b** Number of CTCF clusters at varying clustering window. Starting with a window of 1 bp yields 61,079 mono-plets and using a 10^8^ bp window yields as many clusters as chromosome arms. Shuffled CTCF sites along the genome (blue) are compared to the real spatial distribution of sites (orange). The red line shows the 25-kb clustering window. **c** For each set of clusters composed of 2, 3 and 4 binding sites and subsequently stratified by their class, we show the distribution of their size (distance from the most upstream to the most downstream binding site). The brackets show the *p* values obtained in Bonferroni-corrected *t* tests. **d** Schematic representation of the clustering and pattern finding process. Clusters are non-overlapping sets of adjacent CTCF binding sites and can be decomposed in their various sub-patterns. In particular, in this representation, given the indicated clustering window, we find three clusters of CTCF sites. We also show that cluster 3 corresponds to 4 mono-plets, 3 di-plets, 2 tri-plets and 1 tetra-plet. **e***p* values calculated with the Pearson chi-square test (see Fig. 2) as a function of the clustering window size used in panel **f**. **f** Overrepresented (red) and underrepresented (blue) occurrences of CTCF patterns as a function of the indicated clustering window sizes. The colour scale represents log10 enrichment values (see the ‘Methods’ section). CTCF orientation patterns are ordered by class, as in Fig. 2

The genome-wide median inter-CTCF distance is 21.6 kb (Fig. 3a). However, the distribution of inter-CTCF distances is not uniform as there are more than expected in the 1- to 100-kb range (Fig. 3a, b) as indicated by a *p* value that vanishes to zero in the Mann–Whitney *U* test. To investigate this more closely, we defined the *size* of a CTCF subsequence as the number of base pairs between its most upstream and downstream CTCF site. We hypothesised that the four different pattern classes outlined on Fig. 2 would not show the same size distribution along the chromosomes. Indeed, while ‘Same’ and ‘Conv.+Div.’ tri-plet patterns have very similar size distributions (*p* value = 1.0), convergent tri-plets tend to be significantly larger than divergent patterns (*p* value = 9.8e−55) (Fig. 3c).

Next, we decided to analyse how many patterns can be found if we impose a limit to the inter-CTCF distance between the binding sites composing them. Therefore, given maximal inter-CTCF distance *d*, we aggregated adjacent CTCF sites in clusters (Fig. 3d). Plotting the *p* values from the Pearson chi-square test that we had also used for the chromosomal CTCF patterns at infinite window size (Fig. 2) as a function of the clustering window size shows that the most significance unbalances take place in the 4- to 50-kb range (Fig. 3e). There, *p* values range from non-significant for windows below 4 kb to 10^−45^ for CTCF tetra-plet patterns at the 32.8-kb window. This is in full accord with the primary observation that there are more short-inter-CTCF distances than expected in this size range (Fig. 3a, b). In order to demonstrate that this concerns an overrepresentation of divergent CTCF sites, as suggested by the analysis of pattern sizes (Fig. 3c), we then counted the genome-wide occurrence of each *n*-plet pattern strictly contained within each cluster and divided this by its expected number of instances, defined as the sum of occurrence of all *n*-plet patterns divided by their possible permutations (4 for di-plets, 8 for tri-plets and 16 for tetra-plets). We performed this analysis for logarithmically increasing distances ranging from 4 kb to 268 Mb (Fig. 3f, see the ‘Methods’ section). This revealed that for any class (Same, Conv., Div. and Conv.+Div.) CTCF patterns yield very similar spatial distribution enrichments, which are, however, different between classes. Same orientation CTCF clusters tend to be more prevalent, and Conv.+Div. patterns are depleted, in accord with the fact that Same patterns are more prevalent than Conv.+Div. patterns at the genome-wide level (see Fig. 2). Strikingly, however, convergent and divergent patterns, which obligatorily alternate and are therefore locally as well as globally present in equal numbers (Fig. 2), show opposite spatial distributions between 5 and 131 kb (Fig. 3f); while convergent patterns are depleted in windows up to 100 kb, divergent patterns are enriched in those windows (Fig. 3f).

To test the robustness of this result, we exploited the CTCF aggregate rank score (Additional file 1: Fig. S1) to partition CTCF sites into 4 quartiles which were successively removed prior to re-computation of spatial CTCF distribution density maps (Additional file 1: Fig. S5). This revealed that the enrichment of CTCF patterns in different window sizes was robust to removal of the weakest 25% of sites (Additional file 1: Fig. S5A). Retaining only the top 50% CTCF sites also largely preserved the spatial density imbalances, except for long-range (10^5^ to 10^6^ bp) Same CTCF patterns which became depleted (Additional file 1: Fig. S5B). Finally, ignoring the top 25% of CTCF sites largely preserved the depletion of short-range (10^3^ to 10^5^ bp) convergent patterns, indicating that CTCF clusters made of the lowest three quartiles of CTCF sites still follow the same global spatial distribution imbalances as those made with the 25% top-ranking CTCF binding sites (compare Additional file 1: Fig. S5C to S5F), suggesting a high level of embedding of the divergent CTCF pairs at the expense of convergent CTCF pairs in the distance regime below 100 kb.

In summary, our spatial distribution analyses, which we conducted independently of TAD and gene features, lead us to conclude that CTCF site cluster size distributions along the length of chromosomes are not random with respect to relative CTCF site orientations. Rather, they are hard-wired by CTCF sites belonging to the top three quartiles of CTCF site strength strata, whereby convergent CTCF pairs tend to be more prevalent at long distances (> 100 kb) while divergent CTCF sites are more prevalent at intermediate distances (< 100 kb), and every statistical approach confirms the robustness of this observation.

### Generating a gradient of boundary identity

**Fig. 4:**
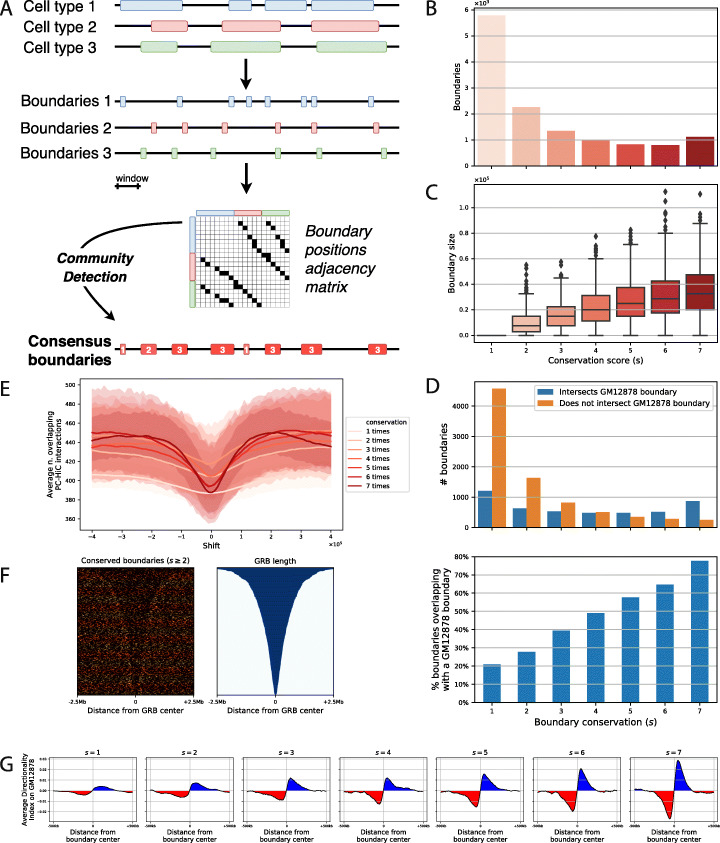
Conserved boundaries show a gradient of enrichment of Hi-C features. **a** Schematic representation of the boundary consensus algorithm using an example of three cell types and a total of ten TADs that yields eight boundaries with conservation scores 1 to 3. For each conservation score, we report **b** the number and **c** the size distribution obtained when starting with the seven primary blood cell type TAD datasets of Javierre et al. [12]. **d** For each conservation score, the number (top) and proportion (bottom) of consensus boundaries that intersect a GM12878 cell boundary are shown. **e** Average number of PC-HIC loops from Javierre et al. [12] that span consensus boundaries of each conservation score. At the centre, the real boundary set is shown and then the coordinates are shifted to the left and right in steps of 10 kb up to 400 kb. **f** Density of consensus boundaries conserved in at least two cell types (s = > 2) on Genomic Regulatory Blocks (GRB) obtained from Harmston et al. [41], aligned at the centre of 5-Mb regions and ordered from largest to smallest. **g** Average directionality index in 5-kb windows, computed on GM12878 and projected onto the s1 to s7 boundaries in 1-Mb windows

In order to dissect the mechanistic roles of CTCF site clusters at boundaries, we needed to identify bona fide boundaries. Hereto, we mined Hi-C TAD boundaries reported by the Blueprint consortium for seven adult human primary blood cell types (erythrocyte, megakaryocyte, macrophage, monocyte, naïve B cell, naïve CD4 T helper cell and naïve CD8 T killer cell) [12] that had been computed using the directionality index (DI) first reported by Dixon et al. [11] at 25-kb resolution. To find consensus boundaries, we developed a ‘boundary consensus’ algorithm. The algorithm takes as input a set of boundaries for each cell type and outputs a unique consensus set of boundaries together with a conservation score for each boundary reporting the number of cell types in which it was detected. Figure 4a visually details this procedure (see the ‘Methods’ section).

The algorithm detects 13,131 boundaries; 5786 of these are ‘cell type-specific’ boundaries in that they have a conservation score of one (s1), while 7345 boundaries are conserved in two or more cell types (s2-s7). Importantly, the results we obtained suggest that the degree of conservation of the boundaries is robust to the number of considered cell types, since the number of boundaries with a conservation score of 4, 5 or 6 is individually less numerous than the boundaries detected in all seven cell types (s7, Fig. 4b). Together, conserved boundaries (s2-s7) occupy 152 Mb of the human genome. The most conserved are the largest, with a median size of 32.5 kb (Fig. 4c, Additional file 4: Table S3).

To independently validate the robustness of this set of boundaries, we processed GM12878 cell line Hi-C data [13] and found 6447 boundaries. Then we checked if consensus boundaries are also boundaries in GM12878 cells. Overall, 20% of the s1 boundaries intersect a GM12878 boundary (see the ‘Methods’). This ratio increases linearly up to 80% for the s7 boundaries (Fig. 4d). Hence, our highly conserved boundaries indeed have a high chance of existing in other human cell types.

To assess boundary function in promoter-bound chromatin looping, we took the approach of Schoenfelder et al. [28] and generated ‘insulation plots’ at the hand of 723,600 chromatin interactions obtained by promoter capture Hi-C (PC-HiC) in the human blood cell types studied by Javierre et al. (see the ‘Methods’ section, [12]). The result shows that boundary insulation capacity is indeed correlated with the consensus score with boundaries s4 to s7 showing strong insulation potential (Fig. 4e).

As a third independent confirmation of boundary function, we projected the s2-s7 boundaries on a set of 815 Genomic Regulatory Blocks (GRBs) known to be conserved throughout vertebrate evolution [41]. The consensus boundaries are clearly enriched at the borders of many of these regions, although some GRBs are interrupted by a boundary, possibly indicating evolutionarily conserved regulatory architectures that involve two or more adjacent TADs (Fig. 4f).

The genomic site of negative to positive sign inversion of the directionality index [11], which indicates a transition from a left to right interaction bias in an Hi-C map, is one of the landmarks used to call HiC-based TAD boundaries [7]. Thus, as a fourth test of boundary identity, we plotted the directionality index of GM12878 cells computed at 25-kb resolution in windows centred on the boundaries of each conservation level (s1-s7). This shows that as boundary conservation increases, the directionality index increases too, following a quasi-linear trend. Hence, the average sharpness of the boundaries increases as a function of their conservation (Fig. 4g). The boundary consolidation score appears to provide a robust metric of boundary strength, since both the insulation [42] and boundary score [43] metrics increase linearly as a function of boundary identity (Additional file 1: Fig. S6).

Altogether, the above results demonstrate that it is possible to stratify boundaries based on their conservation across blood cell types and that features derived from external Hi-C datasets, regulatory interactions and evolutionary conservation can be qualitatively and quantitatively assessed as a function of boundary conservation, therefore defining a TAD boundary ‘identity’ gradient.

### Divergent CTCF site patterns are increasingly enriched at high consensus boundaries

**Fig. 5:**
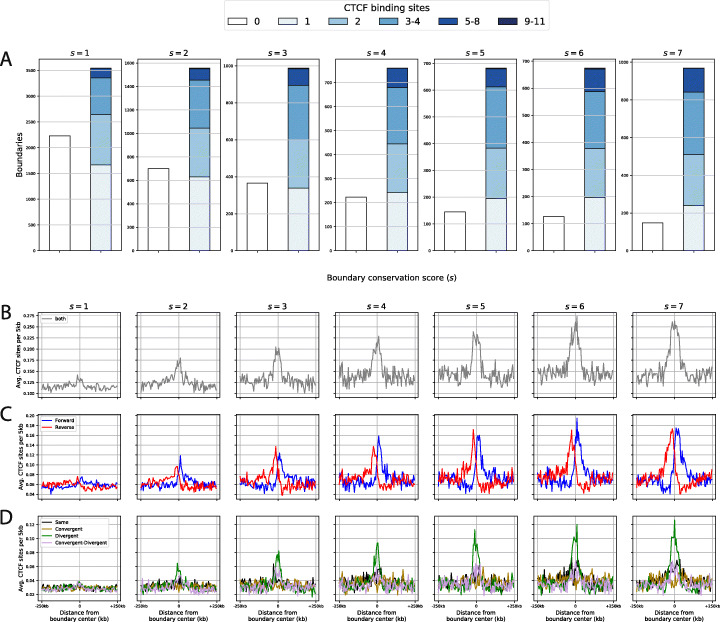
Consensus boundaries are enriched in divergent CTCF sites. **a** Number of boundaries with a given conservation score that harbour 0 to 11 CTCF sites. To circumvent length biases, each boundary was defined by taking its centre and extending that for 25 kb in both directions. **b**–**d** Average number of **b** any CTCF sites, **c** Forward (>, blue) and reverse (<, red) or **d** tri-plet orientation classes shown in 5-kb bins spanning a 500-kb window around the boundary centres and stratified by conservation score

Boundary identity is known to be positively correlated with the number of CTCF sites inside boundaries [11, 13, 34, 44], and this is also true when intersecting our 7345 s2-s7 Blueprint consensus boundaries with the 61,079 ENCODE CTCF sites (Fig. 5a). This supports the notion that CTCF sites encode boundary function. Nevertheless, 16% of our s2-s7 boundaries lack a CTCF site, even when we scanned an additional 25 kb to the left and to the right of the boundary’s borders. However, the proportion of boundaries without CTCF sites decreases from 39% (s1) to 4% for s7 boundaries (Fig. 5a). Thus, some boundaries might perhaps exert their function without CTCF, but this would only concern a minority of the highly conserved boundaries.

To obtain insight into mechanistic roles of CTCF site orientations at boundaries we rendered CTCF site density on 500-kb regions centred on the middle of the boundaries (see the ‘Methods’ section). As expected, this revealed enrichment of CTCF sites at boundaries (Fig. 5b). Next, we partitioned CTCF sites into ‘Forward’ (>) and ‘Reverse’ (<) CTCF sites (Fig. 5c). This split the signal observed on panel 5b into two sets, peaking to the left and to the right of the boundaries, demonstrating that reverse CTCF sites (<) tend to locate to the left side of boundaries while forward CTCF sites (>) tend to locate to the right side.

We then analysed the relationship between the previously defined CTCF pattern classes and the conservation of boundaries. To this end, we assigned a class to each of the 61,079 CTCF sites, following the triplet nomenclature (Same, Convergent, Divergent and Conv.+Div., see Fig. 2) that uniquely defines each CTCF site as a function of its left and right neighbours’ orientations. Remarkably, only one of the four triplet classes is enriched at boundaries, namely the divergent class (Fig. 5d). This observation is buttressed by the fact that enrichment of divergent CTCF sites correlates positively with boundary consolidation score, closely following the DI index increase plotted on Fig. 4g. Hence, the more a boundary is easy to detect, the more likely it is to harbour at least one divergent CTCF tri-plet pattern.

These results strongly suggest that ‘boundary function’ is mechanistically conferred by divergent CTCF site patterns, namely <<> and <>>.

### The CTCF anatomy of TADs

The definition of a TAD boundary is a DI sign switch from negative (−) to positive (+), indicating a point where DNA interaction frequency biases sharply shift from a bias to the left to a bias to the right [11] (Fig. 4g). A corollary of this is that, when moving from left to right through a TAD, there will be a (+) to (−) DI sign change somewhere inside every TAD, so as to switch from the right-biased interactions that delimit the left TAD boundary to the left-biased interactions that delimit the right TAD boundary. The position in a TAD where precisely this (+) to (−) DI sign inversion takes place, which we denote as *(negative) inversion site*, may be determined biophysically through chromatin fibre dynamics [45] and genetically through CTCF sites.

**Fig. 6:**
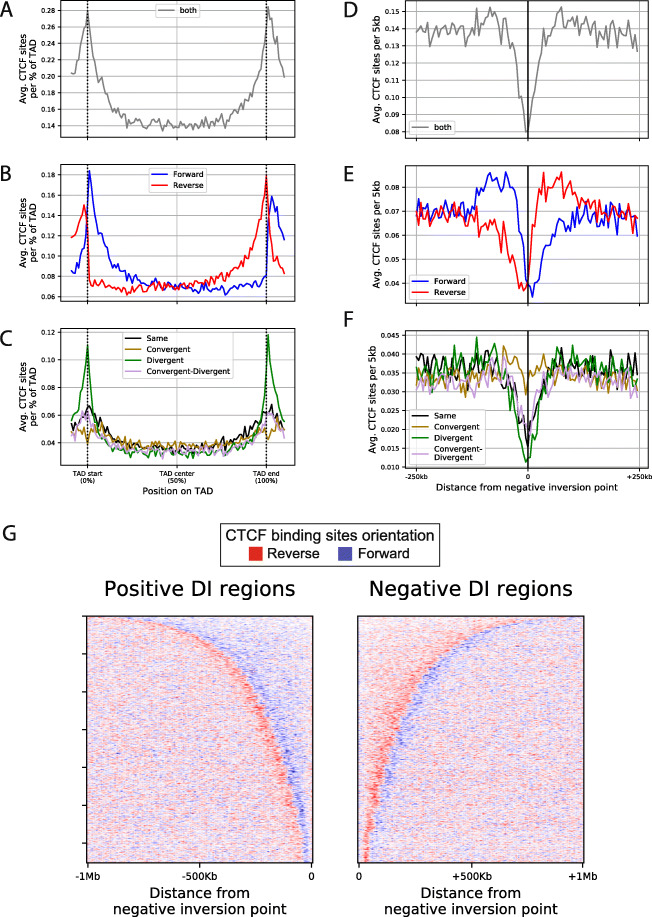
Negative directionality index inversion sites are depleted of three CTCF classes but not the convergent patterns. **a**–**c** Enrichment of **a** CTCF binding sites, **b** CTCF site orientation and **c** CTCF orientation classes along the length of a meta-TAD based on the TAD collection reported by Javierre et al. [12]. Each TAD was divided into 100 bins, and the average number of CTCF sites was computed for each bin. **d**–**f** As for **a**–**c** but for 500-kb windows centred on the negative (+) to (−) points of inversion of directionality index showing **d** the average number of CTCF sites, **e** CTCF site orientation and **f** CTCF orientation classes per 5-kb bins. **g** Distribution of Forward (>, blue) and Reverse (<, red) CTCF sites in 1-Mb window from DI negative inversion points. On the left panel, the positive DI regions extracted from the TADs of GM12878 are ordered by their length from top to bottom and aligned with the negative inversion point to the right. On the right panel, the negative DI regions are ordered by their length from top to bottom and aligned with the negative inversion point to the left. Note that TADs are in general not symmetric with respect to their negative inversion point, as positive and negative DI regions in general have different lengths (see also Additional file 1: Fig. S9A-C)

We first investigated the possibility that CTCF sites determine TAD inversion sites using the 7345 s2-s7 boundaries. In particular, from this collection of consensus boundaries, we computed a set of TADs. To study the general properties of this set of TADs, we then built a ‘meta-TAD’ representation, by projecting their regions to a normalised scale (see the ‘Methods’ section). The meta-TAD shows a cline of Forward (>) CTCF sites on the left half of the meta-TAD. Conversely, the right half shows a cline of Reverse (<) CTCF sites (Fig. 6a–c). This suggests that the DI sign inversion site inside TADs coincides with a reversal in bias of CTCF site orientations.

To further investigate this, we inspected the regions around (+) to (−) DI sign inversion sites of GM12878 and plotted surrounding CTCF site densities (Fig. 6d–f). This revealed that these regions are depleted of same, divergent, and convergent + divergent patterns. In contrast, the density of convergent patterns is preserved at 0.035 per 5 kb (Fig. 6f). Operationally, this results in a relative enrichment of convergent patterns (><<, >><) in a CTCF-depleted local context.

Next, we investigated CTCF site orientation inside left and right TAD ‘halves’ which we defined as the TAD parts located, respectively, to the left and to the right side of negative DI sign inversion sites. We visualised CTCF site orientation on all left- and right-hand TAD ‘halves’ separately, having aligned them on inversion sites and ranked from longest to shortest (Fig. 6g, Additional file 1: Fig. S9A-B). These plots highlight three salient features of the CTCF anatomy of TADs. Firstly, TAD boundaries indeed correspond to CTCF-rich genomic regions that harbour divergent CTCF site arrays flanking TAD boundaries’ central coordinates. Secondly, inversion sites inside TADs often coincide with a reversal of CTCF array directionality and a general depletion of CTCF binding sites with the exception of convergent patterns (Fig. 6f, Additional file 1: Fig. S9A). Thirdly, TAD ‘halves’ are not necessarily of the same size (Additional file 1: Fig. S9B-C) and they may therefore better be referred to as TAD ‘segments’ or ‘sections’. Often, the position of the DI negative inversion point inside TADs is asymmetric (Additional file 1: Fig. S9B).

Hence, the CTCF anatomy of TADs’ left and right sections seamlessly joins boundary CTCF anatomy, since boundaries are enriched in divergent CTCF site arrays (Fig. 5d) that point towards the bordering TADs interiors and converge onto the inversion site that delimits left and right TAD sections.

### Epigenetic marks associated with gene expression are enriched at TAD boundaries

To investigate the relation between TAD boundaries and nucleosome post-translational modifications associated with gene expression, namely H3K4me3, H3K4me1 and H3K27ac, we projected the density of these epigenetic marks [4] onto the size-ranked TAD sections that had been aligned on the inversion site (Additional file 1: Fig. S7A-D). This revealed that there is an enrichment of epigenomic signals at TAD boundaries, consistent with the observation that CTCF sites are often located in the close vicinity of gene promoters (Additional file 2: Table S1, Fig. 1a) and that CTCF sites are enriched at TAD boundaries (Fig. 6g). Hence, as observed by others [11, 13], also in the present analysis, TAD boundaries and epigenomic features known to be associated with gene transcription processes often co-occur.

### Intragenic CTCF sites, TAD boundaries and gene expression

Healthy blood donor monocyte-derived macrophages collectively express almost 15,000 genes above 1 RPKM [4]. More than half of these harbour at least one CTCF site (Additional file 1: Fig. S10A). However, intragenic CTCF cluster site classes do not appear to correlate with transcription direction, since the four CTCF cluster orientation classes outlined on Fig. 2 are similarly represented (Additional file 1: Fig. S10B). This parallels a global lack of correlation between promoter orientation (positive or negative strand) and the 8846 CTCF site’s orientation classes at the 7747 promoter regions of human protein-coding genes that are equipped with a CTCF site (Fig. 1, Additional file 1: Fig. S8, Additional file 2: Table S1). We therefore conclude that CTCF orientation classes are not strictly related to gene orientation along the length of chromosomes.

Stratification of CTCF site strengths into four quartiles (see the ‘Methods’ section, Fig. 1, Additional file 2: Table S1) shows that slightly more 1st than 2nd, 3rd or 4th quartile CTCF sites reside inside transcription units (Additional file 1: Fig. S10C). We therefore infer that strong CTCF sites are not in opposition of gene transcription. Indeed, when scrutinising TAD boundaries, we find that the highest proportion of genes overlapping TAD boundaries concerns the 6201 genes expressed between 10 and 100 RPKM in macrophages, including many boundaries observed in seven primary blood cell types (Fig. 4b, Additional file 1: Fig. S10D). Altogether, the prevalence of CTCF sites and boundaries inside transcribed genes therefore supports the idea that RNA polymerase II can temporarily disrupt any chromatin loop conformation reliant on CTCF.

## Discussion

In the budding yeast lower eukaryote model which lacks a CTCF factor, cohesin is known to accumulate at intergenic regions located between convergent genes, indicating that transcribing RNA polymerase II enzymes can ‘push’ topologically trapped Cohesin complexes up to their polyadenylation sites [46, 47]. In human cells, Cohesin complexes only accumulate at RNA polymerase II polyadenylation sites when CTCF is absent [48]. Otherwise, in the presence of CTCF, Cohesin rings embrace two convergent CTCF sites in more than 50% of the cases [13]. Thus, Cohesin ring localisation can be a by-product of transcription, but CTCF-mediated signals appear to dominate the chromosomal loop landscape through a hypothetical Cohesin-based loop extrusion system that does not require transcribing RNA polymerase II enzymes [36, 37, 48].

Although numerous models explaining the formation of chromatin loops have been proposed [14, 49], a complete theory reconciling the binding of CTCF, the orientation of its motif and the emergence of topological features like TADs and their boundaries was missing.

We find that combining ChipSeq signal and motif affinity measures outperformed either measure of CTCF binding site strength in the context of looping simulations. As an example, the CTCF site strength measures differ in their relationship upstream and downstream of TSS. Therefore, we used an aggregated measure to rank the ENCODE CTCF sites in our study. Then, to elucidate the relationship between CTCF sites and chromatin topological features, every human CTCF site was classified as a function of its own orientation and that of its two neighbours’ orientations.

Considering the tri-plet CTCF pattern nomenclature from a theoretical perspective indicates that Same (>>>, <<<) and Conv.+Div. (<><, ><>) CTCF tri-plets do not change the polarity of the CTCF system, in that those CTCF site’s left and right neighbours have the same orientation as each other. Hence, along any DNA segment, the relative number of Same and Conv.+Div. CTCF sites is variable and can range from none to all and their abundance is independent of the other classes of tri-plets. By contrast, there are strictly as many (± 2) Conv. (>><, ><<) as Div. (<>>, <<>) tri-plet CTCF sites on any one piece of DNA, because convergent and divergent sites must operate in alternating fashion, respectively to change the orientation of the CTCF system from right to left-pointing and from left- to right-pointing. We propose that it is this mathematical property of obligate alternation of Conv. and Div. tri-plets that underlies the emergence of chromosome loop network organisation into topologically associating domains.

In the human genome, same-oriented CTCF patterns are generally enriched across all clustering distances, in keeping with the fact that they are the most prevalent class of patterns. Divergent patterns are most prominent in the short-medium range of 5- to 100-kb inter-CTCF distances, while convergent patterns are depleted at those distances. These results suggest that convergent and divergent patterns operate, respectively, at the TAD level and at the TAD boundary-level size regimes.

By mapping each CTCF tri-plet class to a gradient of boundary identity based on human blood cell TAD boundary conservation, as computed from a consensus algorithm, we demonstrate that TAD boundaries are indeed specifically enriched in divergent CTCF patterns, as had been proposed for the Six homeobox gene cluster [50].

TAD boundaries are defined as sites of abrupt direction change in the polarity of DNA–DNA interactions whereby the directionality index switches from a negative (towards the left) sign to a positive (towards the right) sign [11]. Here, we show for the first time that loci harbouring the converse directionality index sign inversion, from a right-pointing (+) to left-pointing (−) DI index, are depleted of Same, Div. and Conv.+Div. CTCF sites, but retain Conv. CTCF sites.

Generally, therefore, a TAD can be represented as <>(>…>)(<…<)<> with divergent CTCF sites at TAD boundaries and the interior of the TAD harbouring arrays of a variable number of CTCF sites that point to the right (>) in left-hand TAD sections and to the left (<) in right-hand TAD sections.

The extreme statistical significance of the imbalances between the spatial distributions of convergent and divergent CTCF configurations is robust even to the removal of the 25% strongest CTCF sites. This suggests that there is evolutionary pressure to maintain the relative CTCF orientations that form the grammatical rules we discovered, in keeping with the conclusions drawn from CTCF site orientation conservation in syntenic regions of mouse and man [22].

## Conclusions

Altogether, we discovered that, when classified properly, CTCF site orientation forms a powerful grammar that is compliant with recently published loop extrusion models [36, 37].

Hence, our analyses indicate that the two types of directionality index sign inversion points that delimit left and right TAD sections can be hard-wired by combining CTCF site density and CTCF cluster orientation patterns.

In general, chromatin loops and TADs are not completely overlapping because the former are the ‘building blocks’ of the latter. TAD features should be considered as a type of continuant while loops are a type of occurrent. In other words, in any one cell, only one loop (extrusion) conformation can exist at any one point in time, while TAD-level features represent the outcome of the observation of a near infinity of cellular chromosome loop combinations. In this light, the present conciliation of CTCF site orientation and TAD structure has deep implications for the further discovery, study and engineering of chromatin boundaries and TADs. We expect that the mechanistic rules we report here will also be useful to define TAD and TAD boundary models that should eventually yield a comprehensive atlas of human chromosome TADs, sub-TADs and boundaries.

## Methods

### Assigning scores to CTCF binding sites and motifs

For our analysis, we used the CTCF binding sites from GM12878 and used their motif orientation as calculated in Rao et al. [13]. We refer to a motif as ‘right’ (>) when it is present on the forward strand of the chromosome and ‘left’ (<) when it is on the reverse strand. We excluded from the analysis all binding sites without any motif match.

We then downloaded the 33 ENCODE Narrow Peak tracks for CTCF in different cell lines and growth conditions as well as primary human tissues (Additional file 3: Table S2) from the UCSC Browser Open Chromatin Transcription Factor Binding Sites table [33]. For each CTCF binding site in our initial set from Rao et al. [13], we then associate the reported enrichment signal (*signalValue*) for each of the Chipseq tracks, using the *map* operation of PyGMQL [51], taking the maximum value in case more than one peak is mapped on the same binding site. Before aggregating the 33 signal values for every CTCF binding site, we assessed the value distribution of every CTCF Chipseq experiment and found that the distributions across cell lines, lineages and laboratories were heterogeneous (Additional file 1: Fig. S11). To use comparable input values before computing the binding site Chipseq scores, we performed a quantile normalisation across all the experiments. For each CTCF binding site, we then summed up the signal of the 33 ENCODE tracks; we call this the Chipseq score (Additional file 1: Fig. S1C).

HOMER motif calling software was used to assess motif quality at each binding site [52]; we used the CTCF consensus sequence MA0139.1 from JASPAR [53] and used the findMotifsGenome.pl function to extract the matching score for the best motif instance at each binding site (Additional file 1: Fig. S1B).

Motif and Chipseq scores have different distributions (Additional file 1: Fig. S1B-C), as the former has a normal-like distribution while the latter has a Poisson-like distribution; we multiplied their ranks so as to take into account both contributions.

### Loop extrusion simulation and validation of CTCF scores

To simulate loop extrusion and evaluate the goodness of motif, ChipSeq and rank aggregated score, we first assigned to each CTCF binding site a *permeability* score, which is a number between 0 and 1 describing the probability of the extrusion complex to ignore a correctly oriented CTCF site during the pulling phase [14]. This number is dependent on the selected score as explained below.

We first designed a ‘full-stop’ model in which all the CTCF sites have a permeability score of 0, meaning that the extrusion complex will always stop pulling DNA when it encounters a correctly oriented CTCF motif (Additional file 1: Fig. S1B-C—blue lines). We then considered a set of models based respectively on ChIPseq, motif and rank score. For each CTCF site *c*, we defined its permeability score *v* in these models using min-max normalisation as follows:

$$ {\displaystyle \begin{array}{c}{v}_c^{\mathrm{full}\ \mathrm{stop}}=0\\ {}{v}_c^{\mathrm{ChIPseq}}=1-\frac{s_c^{\mathrm{ChIPseq}}-\min \left({s}^{\mathrm{ChIPseq}}\right)}{\max \left({s}^{\mathrm{ChIPseq}}\right)-\min \left({s}^{\mathrm{ChIPseq}}\right)}\\ {}{v}_c^{\mathrm{Motif}\ \mathrm{score}}=1-\frac{s_c^{\mathrm{Motif}\ \mathrm{score}}-\min \left({s}^{\mathrm{Motif}\ \mathrm{score}}\right)}{\max \left({s}^{\mathrm{Motif}\ \mathrm{score}}\right)-\min \left({s}^{\mathrm{Motif}\ \mathrm{score}}\right)}\\ {}{v}_c^{\operatorname{rank}\ \mathrm{score}}=1-\frac{s_c^{\operatorname{rank}\ \mathrm{score}}-\min \left({s}^{\operatorname{rank}\ \mathrm{score}}\right)}{\max \left({s}^{\operatorname{rank}\ \mathrm{score}}\right)-\min \left({s}^{\operatorname{rank}\ \mathrm{score}}\right)}\end{array}} $$

For each simulation epoch, we then executed an extrusion event between each pair of two adjacent CTCF binding sites. Our simulations are therefore independent of the CTCF density fluctuations along the genome. The hypothetical extrusion complex pulls DNA on its right and left at the same time and independently stops extrusion on each side when the following two conditions apply: firstly, the complex has reached a convergent CTCF site and, secondly, a uniformly generated number between 0 and 1 is lower than the permeability score of this CTCF site. Note that the same loop can be generated multiple times in the same epoch and across epochs. In this way, we can model both the statistical variability of loop formation due to CTCF permeability and the geometrical constraints due to CTCF orientation of spatial patterns. We fixed the number of epochs to 100 and parallelised each simulation epoch, to speed up the execution.

To evaluate how much the various simulations were able to recall known topological features, we downloaded high confidence long-range loops in GM12878, as extracted by the HiCCUPS algorithm from Rao et al. [13]. We then selected only the HiCCUPS loops having one pair of convergent CTCF motifs at their two anchors. We then computed the percentage of HiCCUPS loop (recall) that are recovered by the various simulation models as a function of the number of times they occurred in the simulations (Additional file 1: Fig. S1E). Precision–recall curves of the indicated models were used to investigate the percentage of simulated loops that are present in the HiCCUPS collection as a function of recall (Additional file 1: Fig. S1F).

### Properties of the CTCF subsequences

Thanks to our CTCF pattern classification, we can observe a simple mathematical rule governing sequences of CTCF binding site on any stretch of DNA, generalising therefore to the whole genome. Given a sequence of *N* CTCF binding sites, the number of patterns of length *K* is *N − K + 1* (61,079 for mono-plets, 61,037 for di-plets, 60,995 for tri-plets and 60,953 for tetra-plets). In our analysis, we discarded chromosome Y as not all donors possess it. Furthermore, the 61,079 Encode collection of CTCF sites misses the p-arms of the acrocentric human chromosomes 13, 14, 15 and 22, leaving us with 42 chromosome arms.

In our genome-wide calculation (Fig. 2), we therefore divide the genome in 42 sequences of CTCF sites using the chromosome arms and their centromeres as division points. Therefore, the maximal skews for divergent and convergent patterns are 42 for di-plets, 84 for tri-plets and 126 for tetra-plets.

On any one piece of DNA, the sum of either Same and Conv.+Div. CTCF tri-plets can vary from 0 to the sum of all CTCF sites minus two, considering the CTCF sites located at the ends of the DNA molecule. On the other hand, there will always be as many divergent as convergent CTCF di-plet (± 1) and tri-plet (± 2) patterns on any one piece of DNA. This is best exemplified at the level of di-plets. When a CTCF site points to the right, its next neighbour to the right (>) can only be in the same orientation (>) or pointing to the left (<). In the former case, the resulting di-plet is of the class ‘same’ (>>); in the latter case, it is of the class ‘convergent’ (><). Conversely, a CTCF site pointing to the left (<) can only form same or divergent di-plets with its right neighbour (<<, <>). This property is satisfied when considering all possible CTCF sequence patterns in a DNA piece regardless of the distance between the binding sites composing them. However, when inter-CTCF distance is used to cluster CTCF binding sites, the relative number of CTCF patterns can vary.

### Distance of the observed genome-wide CTCF pattern class abundances from a randomised distribution of CTCF orientations

To test if the genome-wide unbalance between pattern classes in bi-, tri- and tetra-plets, we used two statistical testing approaches. We first performed a Pearson chi-square test (*p* values reported in Fig. 2), revealing high significance for all three pattern sizes.

We then considered a more data-driven approach, based on the randomisation of the CTCF site orientations. For di-plets, tri-plets and tetra-plets, we performed the following steps: (1) we calculated the theoretical expected value for the proportions of classes (in the case of di-plets, Same = 0.5, Conv = 0.25, Div. = 0.25; in the case of tri-plets, Same = 0.25, Conv = 0.25, Div = 0.25 and Conv. Div. = 0.25; for tetra-plets, Same = 0.125, Conv = 0.187, Div. = 0.187 and Conv. Div. = 0.5); (2) we randomised the orientations of CTCF sites in the genome (keeping their position fixed) for 10,000 times; (3) we calculated an empirical expected value of the frequencies of each pattern category for each randomisation; (4) we computed the chi-squared statistic

$$ \sum \limits_{c\in \left\{S,D,C, CD\right\}}\frac{{\left({O}_c-{E}_c\right)}^2}{E_c} $$

as a distance measure between the frequency values of each randomisation and the theoretical expected values; (5) finally, we compared the distance between the observed frequencies and the theoretical ones with the previously computed distribution.

In this way, we have a visual and quantitative assessment of the shift of our data, divided by the size of the pattern. All the skews (which can be calculated as empirical *p* values) are very significant, and they all converge to 0 (Additional file 1: Fig. S4).

### CTCF spatial distribution analysis

We designed a clustering procedure of CTCF binding sites based on their distance. Given a maximal inter-CTCF distance *d*, each cluster is composed of a set of adjacent CTCF sites spaced by less than *d* base pairs. We used the *cluster* function of BEDTools [54] to extract the clusters using *d* as maximum distance parameter. Then, for each cluster, we extracted all mono-plets, di-plets, tri-plets and tetra-plets (see Fig. 3d for a schematic representation); the distance between each pair of CTCF sites composing one of these patterns is therefore less than or equal to *d*. This clustering procedure identifies, at varying *d*, how orientation patterns change in relative density along the genome. Notice that if a pattern is identified using a specified clustering window, it is also found at all larger windows. When *d* is equal to the chromosome size, we end up with one cluster for each chromosome arm and with the complete set of *n-*plets, and their counts converge to the numbers reported in Fig. 2.

Given each clustering window *d,* we asked ourselves, for each pattern *p* composed of *n* binding sites, if *p* was over- or underrepresented with respect to the total number of patterns bearing the same number of sites found for *d*. We used as reference an expected distribution assuming that the proportion of occurrences of patterns with the same number of binding sites is uniform for each clustering window *d* (¼ for di-plet, 1/8 for tri-plets and 1/16 for tetra-plets). We finally computed the log10 ratio of observed and expected counts for each pattern at each window (Fig. 3f). Statistical significance across clustering windows was assessed using the Pearson chi-squared test (Fig. 3e).

We performed the above procedure initially with the complete set of CTCF sites. We then divided CTCF sites in four equally populated subsets based on their rank aggregated score, that we call quartiles. Next, we performed the clustering procedure and statistical analysis upon successively removing each quartile (Additional file 1: Fig. S5).

### Boundary consensus algorithm

We developed a novel algorithm to find the consensus regions across several TAD boundary datasets. The algorithm takes as input a set of boundary datasets in the form of genomic regions and a *detection window* (*w*), which determines the sensitivity of the procedure in detecting overlapping boundaries. In our study, given the minimum Hi-C resolution adopted by Javierre et al. [12] of 25 kb, we imposed *w = 25 kb*.

As a first step, the procedure extracts for each boundary of each dataset its *boundary positions*, defined as its start or stop positions. The algorithm builds then an adjacency matrix *A* having as rows and columns all boundary position across all the datasets. A cell is equal to one if the two positions corresponding to its row and column have a distance less or equal than *w*, zero otherwise. Thus *A* is symmetric. We then apply the Louvain modularity [55] community detection algorithm to *A* and find clusters of boundary positions. We used the python-louvain Python package to extract the clusters of boundary positions using the best_partition function with default parameters. It must be noted that the usage of a community detection strategy is not equivalent to simply calling the connected components on the graph defined by *A*. The algorithm instead tries to find clusters which maximise the density of connections between the boundary positions, each corresponding to a set of nearby boundary positions across multiple datasets.

The *consensus region* of a cluster is the genomic region between the most upstream and downstream boundary positions of a cluster. The centre of the computed boundary is halfway. The *boundary conservation score* is the number of cell types from which the region derives (Fig. 4a).

### Intersecting GM12878 boundaries with consensus boundaries

We downloaded the GM12878 ‘primary + replicate combined’ *.hic* file from Rao et al. 2014 [13]. We used the Cooler software [56] for data processing and analysis. Coherently with Javierre et al. [12], we performed iterative correction of the contact matrix [57] and then calculated directionality index [11] at a bin size of 25 kb. Boundaries were called at peaks of insulation score with the cooltools suite.

We then counted how many GM12878 boundaries intersect the consensus boundaries and stratified the results by conservation score (Fig. 4d). Since the length of the consensus boundaries is variable and dependent on their conservation score, we circumvented length biases by taking the centre of each boundary and extending it 25 kb to the left and to the right using BEDTools [54].

### Relationship between PC-HiC interactions, Genomic Regulatory Blocks and consensus boundaries

We downloaded 723,600 promoter capture Hi-C (PC-HiC) interactions with CHICAGO score [58] greater than 5 from Javierre et al. [12]. Next, following the strategy of Schoenfelder et al. [28], we computed the average number of promoter–genome interactions which span each boundary. We computed the same metric using each s1-s7 boundary set (Fig. 4e—centre). Each boundary of each set was then shifted in steps of 10 kb to the left and to the right up to 400 kb (Fig. 4e—left and right points), calculating the same metric as before. In this way, we were able to model the local background of each boundary set, therefore showing its insulation capability.

We then downloaded the dataset of Genomic Regulatory Blocks (GRBs) from Harmston et al. [41]. To determine how our consensus boundaries relate with these highly conserved regions, we ordered GRBs based on their length and aligned them on their centre. We then plotted the position of boundaries conserved in at least 2 cell types in a surrounding 5-Mb window around the centre of each GRB (Fig. 4f) with a resolution of 5 kb.

### The boundary identity gradient identified by the consensus algorithm provides a robust metric of boundary insulation

We validated our gradient of boundary identity also through direct measurement on Hi-C data. In our study, we compared three different boundary scoring methods, namely the directionality index [11] (Fig. 4g), the insultation score [42] (Additional file 1: Fig. S6B) and the boundary score calculated by the TADCompare package [43] (Additional file 1: Fig. S6C). All three scoring methods yielded a linear absolute increase of the reference metric, demonstrating that the identity gradient provided by our consensus strategy is a good metric of ‘border strength’.

### Enrichment analysis of CTCF patterns at consensus boundaries

We counted how many boundaries harbour 0, 1 or more CTCF binding sites. The results were stratified by conservation score (Fig. 5a). Also, in this case, since consensus boundaries have heterogeneous lengths, we first took the centre of each boundary and looked at its 25 kb upstream and downstream regions.

We then assigned each CTCF binding site a class depending on its two adjacent sites. There are therefore eight different possible tri-plet patterns, which we then name following the classification shown in Fig. 2. Note that the classification concerns the central CTCF site of the tri-plet and is independent of the distances between CTCF sites forming the tri-plet. We then took the centres of all consensus boundaries, looked at the region 250 kb upstream and downstream and computed the average number of all CTCF sites (Fig. 5b) and then consider each motif orientation (Fig. 5c) and class (Fig. 5d) in windows of 5 kb.

### Enrichment analysis of CTCF patterns and epigenetic marks on TADs

We investigated the average enrichment of CTCF sites (Fig. 6a), their orientation (Fig. 6b) and their classes (Fig. 6c) on TADs by computing the set of consensus TADs from our collection of 7345 s2-s7 consensus boundaries. TAD regions were defined as the complement of boundary regions, excluding genomic gaps. We then divided each TAD in 100 equal bins and counted how many binding sites fall inside each bin. Note that the size of bins is different for each TAD. We then aggregated all the bin values for each TAD in a single vector having one value for each bin, representing the average enrichment. Therefore, the *x*-axis in Fig. 6a–c can be seen as relative positions, with zero representing the beginning and one the ending of TADs.

We then focused on the central position of TADs. Points of positive inversion (−) to (+) in the directionality index are an indication of abrupt increase of insulation determining a boundary (Fig. 6a–c). The negative inversion sites are smoother and tend to localise towards the middle of TADs. We therefore took the directionality index computed at 25-kb resolution on GM12878 and identified points of negative inversion along the genome. We then looked at the region 250 kb upstream and downstream of each negative inversion point and computed the average number of all CTCF sites (Fig. 6d) and then considering each motif orientation (Fig. 6e) and class (Fig. 6f) in windows of 5 kb.

Each TAD can be seen as the composition of two regions; the first starts at the point of positive inversion and ends at the one of negative inversion (*positive DI*), and the second starts at the point of negative inversion and ends at the one of positive inversion (*negative DI*). We therefore sorted all the positive and negative DI regions found in GM12878 separately, ordering them by their size and aligning them on the negative sign inversion point. The presence of forward and reverse CTCF binding sites was then rendered within windows of 5 kb on the 1-Mb genomic interval spanning each DI region, respectively as blue or red (Fig. 6g, Additional file 1: Fig. S7A, S9A).

We finally downloaded the H3K27ac, H3K4me1 and H3K4me3 epigenetic marks detected in macrophages from Wang et al. [4] and rendered their density within windows of 5 kb, spanning the 1-Mb genomic intervals aligned on the negative directionality index inversion points (Additional file 1: Fig. S7B-D), as shown in Fig. 6g for CTCF site directionality.

## Supplementary information

**Additional file 1: Fig. S1**: CTCF looping simulation performance increases when ChIPseq and Motif scores are integrated. **Fig. S2**: Properties of CTCF binding sites at promotorial regions. **Fig. S3**: DNA methylation analysis of promoter CTCF sites. **Fig. S4**: Distance of the observed genome wide CTCF pattern classes abundances from a randomised distribution of CTCF orientations. **Fig. S5**: Stability of CTCF cluster spatial patterns. **Fig. S6**: The boundary identity gradient identified by the consensus algorithm provides a robust metric of boundary insulation. **Fig. S7**: Density of epigenetic marks in positive and negative directionality index TAD parts. **Fig. S8**: CTCF spatial classes do not correlate with gene orientation. **Fig. S9**: TADs can be divided in asymmetric halves by their directionality index negative inversion point. **Fig. S10**: Relationships between intragenic CTCF sites, TAD boundaries and gene expression. **Fig. S11**: ChIPseq signal distribution of the 33 CTCF Narrow Peak tracks.

**Additional file 2: Table S1.** CTCF binding sites collection and associated annotations.

**Additional file 3: Table S2.** Metadata for the 33 CTCF ChIPseq Narrow Peak datasets downloaded from ENCODE used in this study.

**Additional file 4: Table S3.** Consensus boundary collection generated in this study.

**Additional file 5.** Review history.

## Data Availability

CTCF sites, ENCODE samples we used and the consensus boundaries we generated in this study are available as Additional file 2: Table S1, Additional file 3: Table S2 and Additional file 4: Table S3 respectively. PC-HiC interactions and imputed TAD boundaries from the seven cell lines where downloaded from Javierre et al. [12]. Genomic Regulatory Blocks (GRBs) where downloaded from Harmston et al. [41]. Gene expression quantification of monocyte-derived macrophages was downloaded from Wang et al. [4]. The code that reproduces all the analyses presented in the manuscript is available at GitHub (https://github.com/lucananni93/CTCF_Spatial_Patterns) [59] and Zenodo [60].
