# Supplementary material for: Spatial patterns of CTCF sites define the anatomy of TADs and their boundaries
Source: Genome Biol. 2020 Aug 12;21:197. doi: 10.1186/s13059-020-02108-x (PMC7422557; doi:10.1186/s13059-020-02108-x)
Supplement: Supplementary file 1 — Additional file 1: Fig. S1: CTCF looping simulation performance increases when ChIPseq and Motif scores are integrated. Fig. S2: Properties of CTCF binding sites at promotorial regions. Fig. S3: DNA methylation analysis of promoter CTCF sites. Fig. S4: Distance of the observed genome wide CTCF pattern classes abundances from a randomised distribution of CTCF orientations. Fig. S5: Stability of CTCF cluster spatial patterns. Fig. S6: The boundary identity gradient identified by the consensus algorithm provides a robust metric of boundary insulation. Fig. S7: Density of epigenetic marks in positive and negative directionality index TAD parts. Fig. S8: CTCF spatial classes do not correlate with gene orientation. Fig. S9: TADs can be divided in asymmetric halves by their directionality index negative inversion point. Fig. S10: Relationships between intragenic CTCF sites, TAD boundaries and gene expression. Fig. S11: ChIPseq signal distribution of the 33 CTCF Narrow Peak tracks. [file 13059_2020_2108_MOESM1_ESM.pdf]

# Spatial patterns of CTCF sites define the anatomy of TADs and their boundaries

Luca Nanni, Stefano Ceri and Colin Logie

## *Supplementary Materials*

## Contents

**Fig. S1**: CTCF looping simulation performance increases when ChIPseq and Motif scores are integrated .. 2

**Fig. S2**: Properties of CTCF binding sites at promotorial regions ........ 3

**Fig. S3**: DNA methylation analysis of promoter CTCF sites ........ 5

**Fig. S4**: Distance of the observed genome wide CTCF pattern classes abundances from a randomized distribution of CTCF orientations........ 6

**Fig. S5**: Stability of CTCF cluster spatial patterns........ 7

**Fig. S6**: The boundary identity gradient identified by the consensus algorithm provides a robust metric of boundary insulation ........ 8

**Fig. S7**: Density of epigenetic marks in positive and negative directionality index TAD parts........ 9

**Fig. S8**: CTCF spatial classes do not correlate with gene orientation ........10

**Fig. S9**: TADs can be divided in asymmetric halves by their directionality index negative inversion point 11

**Fig. S10**: Relationships between intragenic CTCF sites, TAD boundaries and gene expression........12

**Fig. S11**: ChIPseq signal distribution of the 33 CTCF Narrow Peak tracks........13

References........14

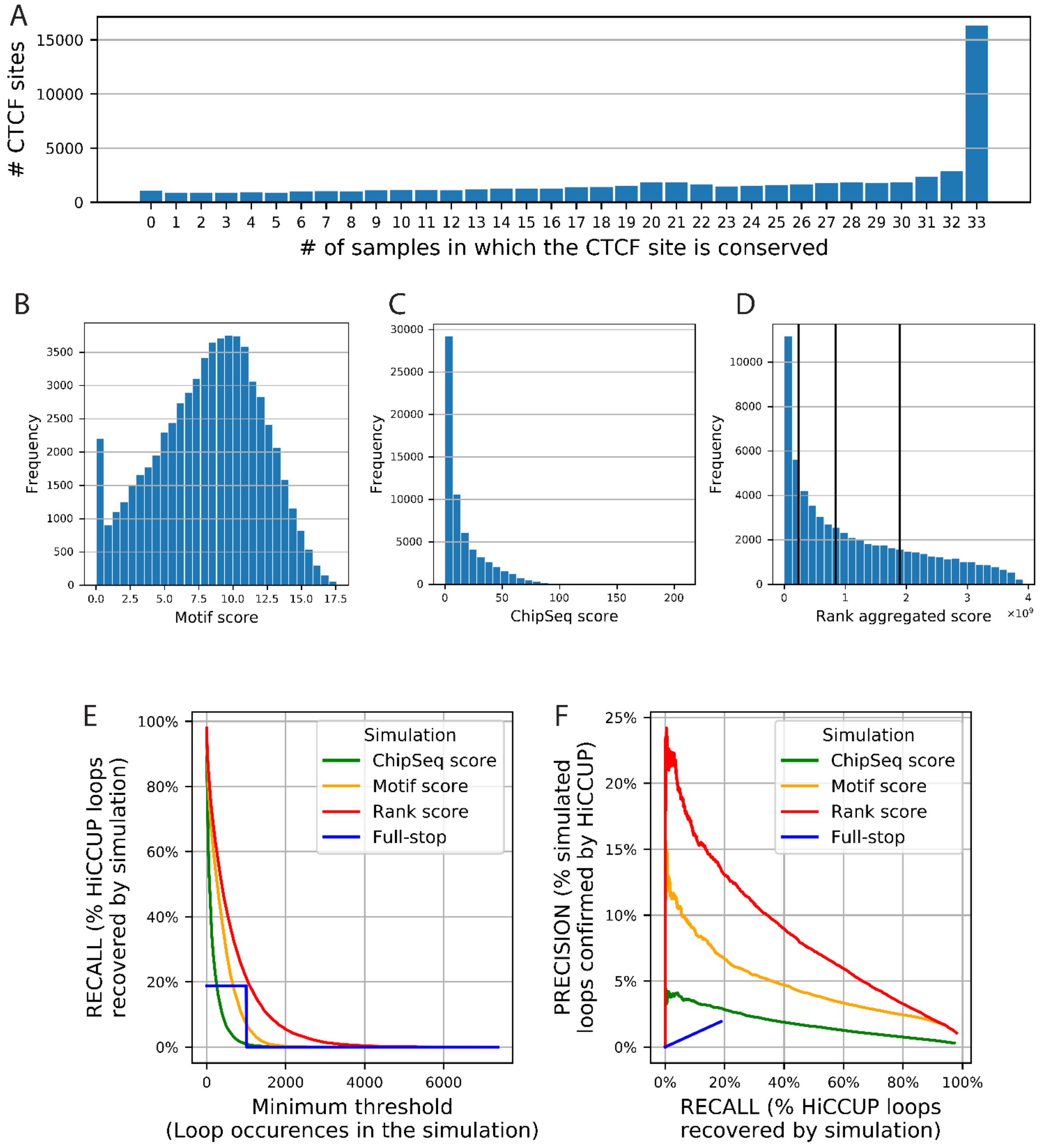


## Fig. S1: CTCF looping simulation performance increases when ChIPseq and Motif scores are integrated

(**A**) Number of CTCF binding sites from Rao et al. 2014 conserved in zero to all 33 ENCODE peak datasets. (**B**) For all CTCF binding sites, value distribution of the motif scores as computed by HOMER [1], (**C**) of the aggregated ChipSeq signal scores and (**D**) of rank aggregated scores. The three vertical black lines in (**D**) indicate the 25%, 50% and 75% quartiles which were used for **Fig. S4A-F** and **S9C**. (**E-F**) Performance of CTCF looping simulations: (**E**) Percentage of HiCCUPS loops [2] that are recovered by the various models (recall) as a function of their occurrence in the simulations; (**F**) Precision-recall curves of the indicated models, indicating the percentage of simulated loops which are present in the HiCCUPS collection as a function of recall.

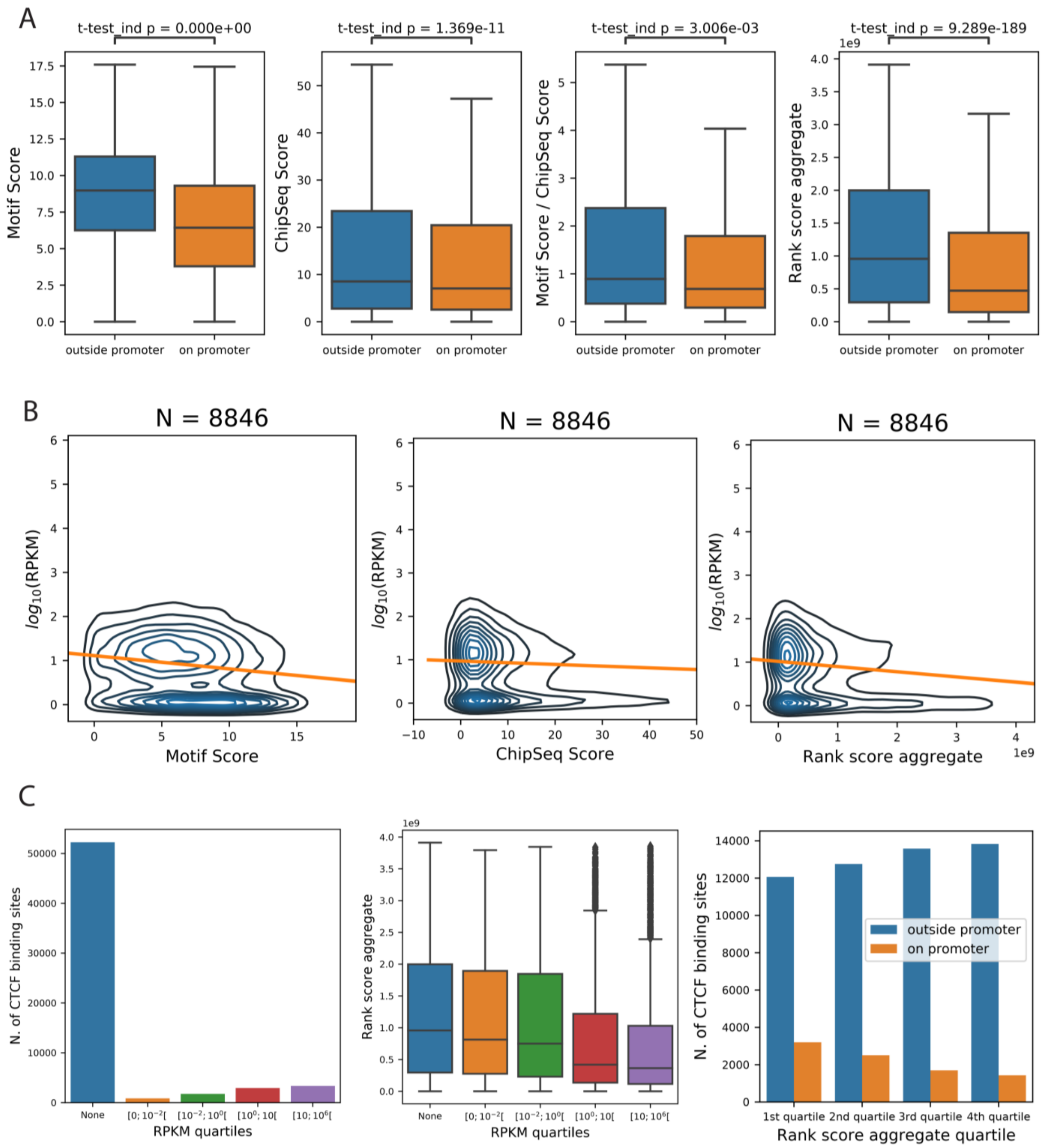


## Fig. S2: Properties of CTCF binding sites at promotorial regions

(**A**) Motif score, ChipSeq score, Motif and ChipSeq score ratio and rank aggregated score for CTCF binding sites outside (blue) and inside (orange) promoter regions (-2 kb to +2 kb from the TSS region of coding genes). (**B**) Kernel density estimation plot displaying the relationship between Motif, ChipSeq and Rank aggregated score and the expression of genes, for the 8,846 CTCF binding sites in 7,747 promoter regions. (**C**) Number (*left*) and rank aggregated score (*centre*) of CTCF binding sites and their overlapping promotorial regions divided by the expression level of their gene; number of CTCF binding sites inside and outside of promoter regions as a function of their rank aggregated score quartile (*right*).

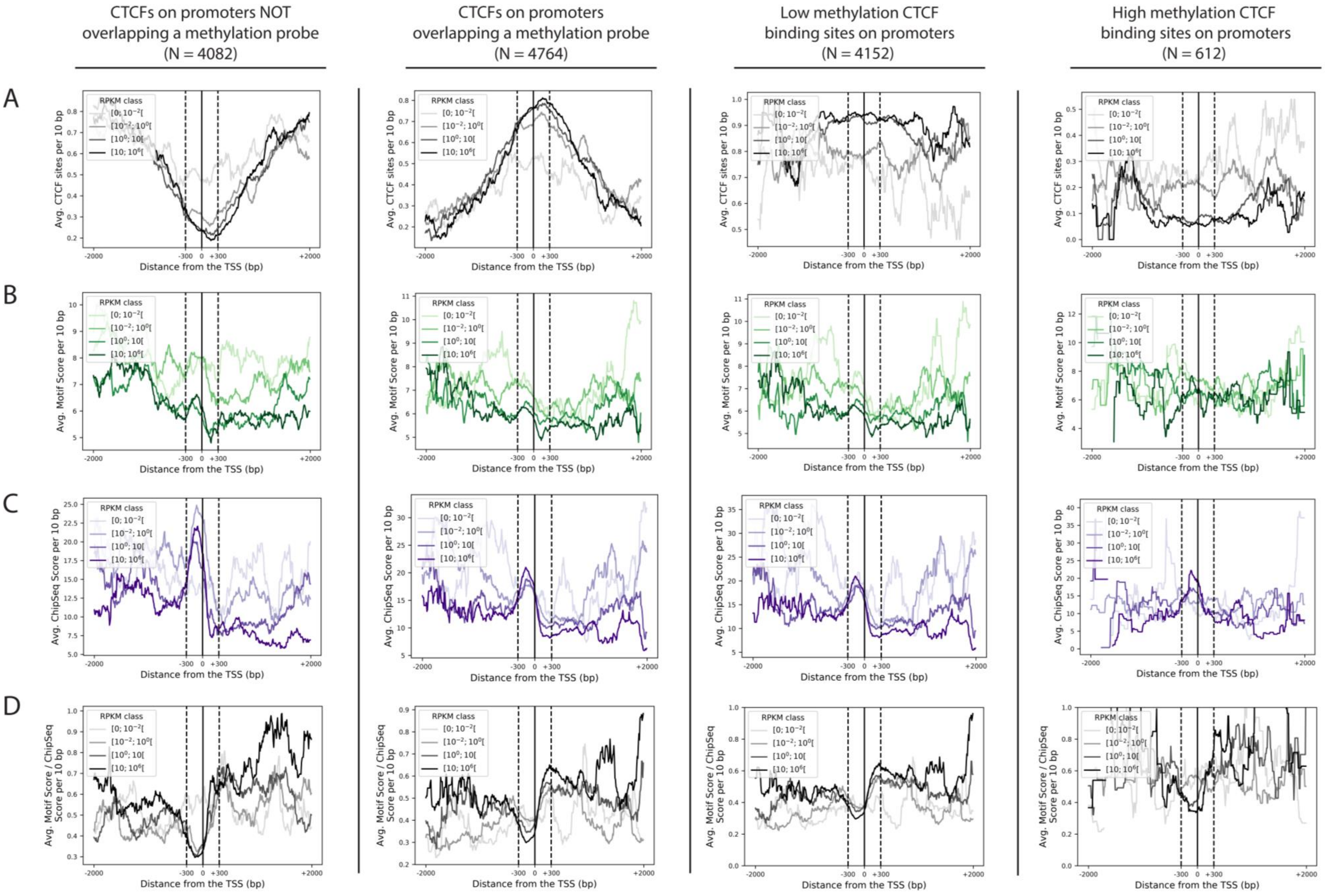
CTCFs on promoters NOT overlapping a methylation probe (N = 4082)
CTCFs on promoters overlapping a methylation probe (N = 4764)
Low methylation CTCF binding sites on promoters (N = 4152)
High methylation CTCF binding sites on promoters (N = 612)
A
B
C
D
Avg. CTCF sites per 10 bp
Avg. Motif Score per 10 bp
Avg. ChipSeq Score per 10 bp
Avg. Motif Score / ChipSeq Score per 10 bp
Distance from the TSS (bp)
RPKM class
[0; 10^−2[
[10^−2; 10^0[
[10^0; 10[
[10; 10^6[
-2000
-300
0
+300
+2000

## Fig. S3: DNA methylation analysis of promoter CTCF sites

Spatial distribution of CTCF motifs located around the transcription start sites of human protein-coding genes without (*left-most*) and with DNA methylation data (*left middle*) and for those with methylation data, separately for CTCF sites with low (*right middle*) and with high (*right-most*) DNA methylation level. DNA methylation data are from Reduced Representation Bi-sulfite Sequencing (RRBS) on the GM12878 cell line DNA (GEO: GSM683841). The data are plotted as a function of gene expression strength quartiles (**A-D**).

Altogether, this analysis demonstrates that low CTCF site methylation is the representative state for the large majority of promoter CTCF sites. Crucially, the unmethylated CTCF binding site population displays the 50% increase in ChIPseq to Motif score ratio when comparing CTCF sites that lie less than 300bp upstream of the TSS to those that lie less than 300 bp downstream of it. Hence, there does not appear to be any ground to conclude that CTCF site CpG methylation confounds the conclusion that the ratios of CTCF motif score to CTCF ChIPseq signal differ according to the CTCF site's location, when active gene promoters are considered (**Fig. 1D**).

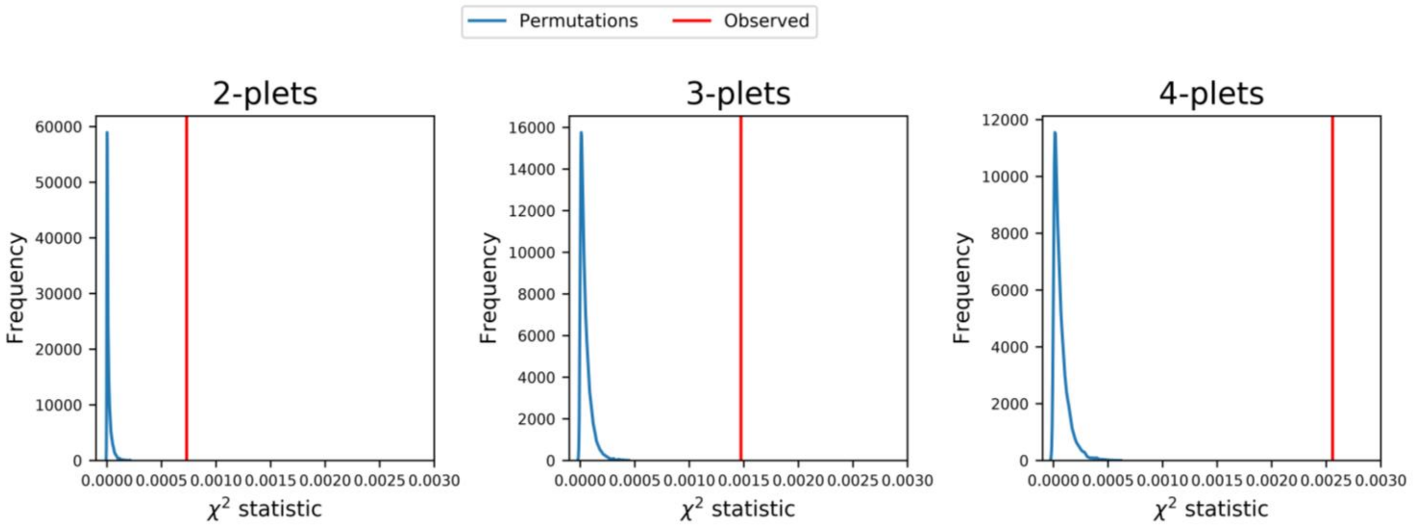


## Fig. S4: Distance of the observed genome wide CTCF pattern classes abundances from a randomized distribution of CTCF orientations

For di-plets (*left*), tri-plets (*centre*) and tetra-plets (*right*) counted in the whole human genome, we report the distance of their CTCF pattern classes counts from a uniform distribution (red lines). The distance is calculated as a Chi-squared statistic (See Methods). We also calculate the distance of 10,000 randomizations of all the genome-wide CTCF orientations from the uniform distribution (blue densities). All empirical p-values converged towards zero.

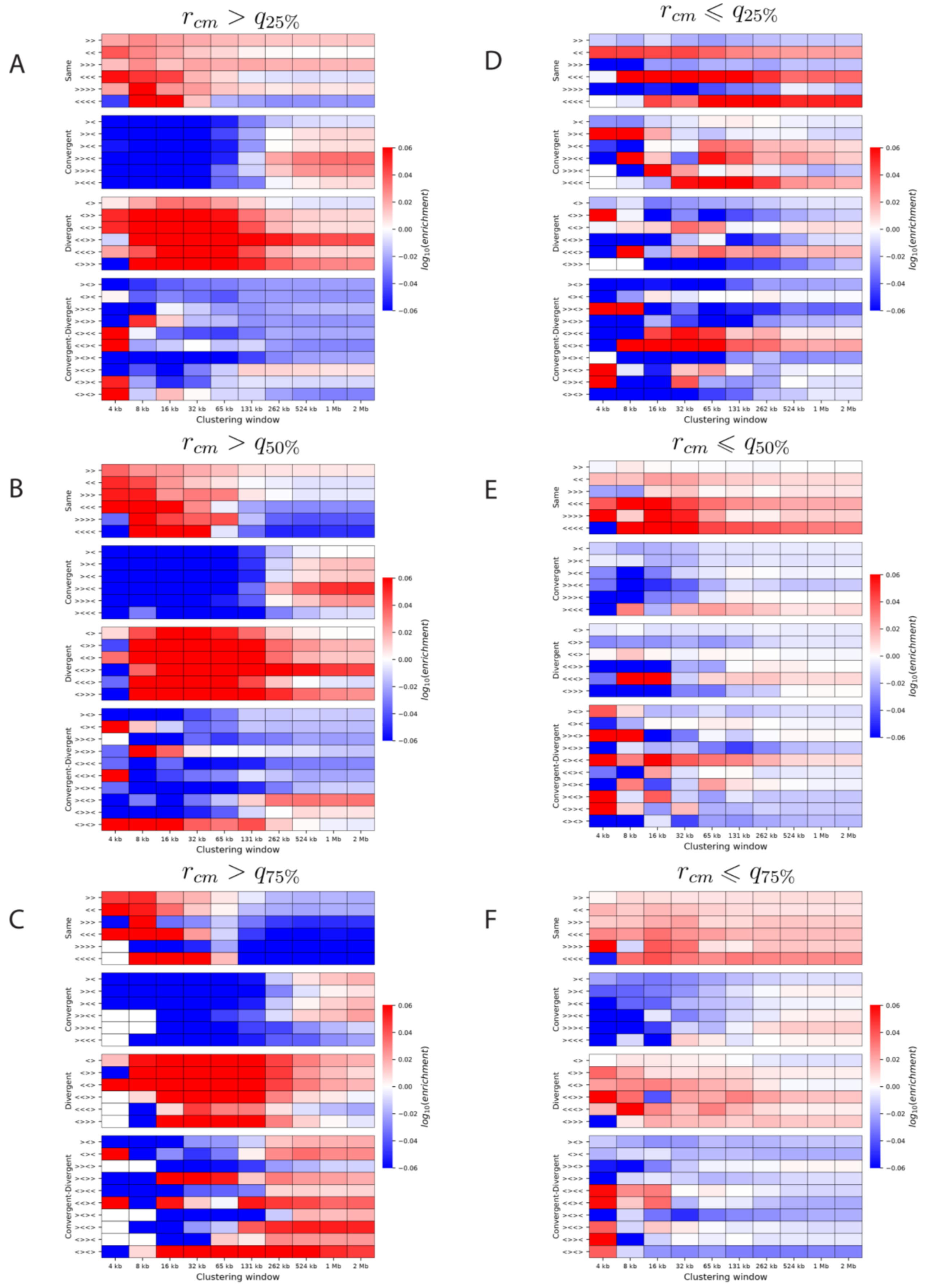


## Fig. S5: Stability of CTCF cluster spatial patterns

Over-represented and under-represented CTCF patterns (plotted as in **Fig. 3F**, see Methods) obtained by successively removing CTCF binding sites in descending (**A** to **C**) and ascending (**D** to **F**) quartiles of the rank score distribution, followed by calculation of the number of instances for each pattern. Each heatmap consists of the quartile intervals of CTCF sites that is indicated above the panel.

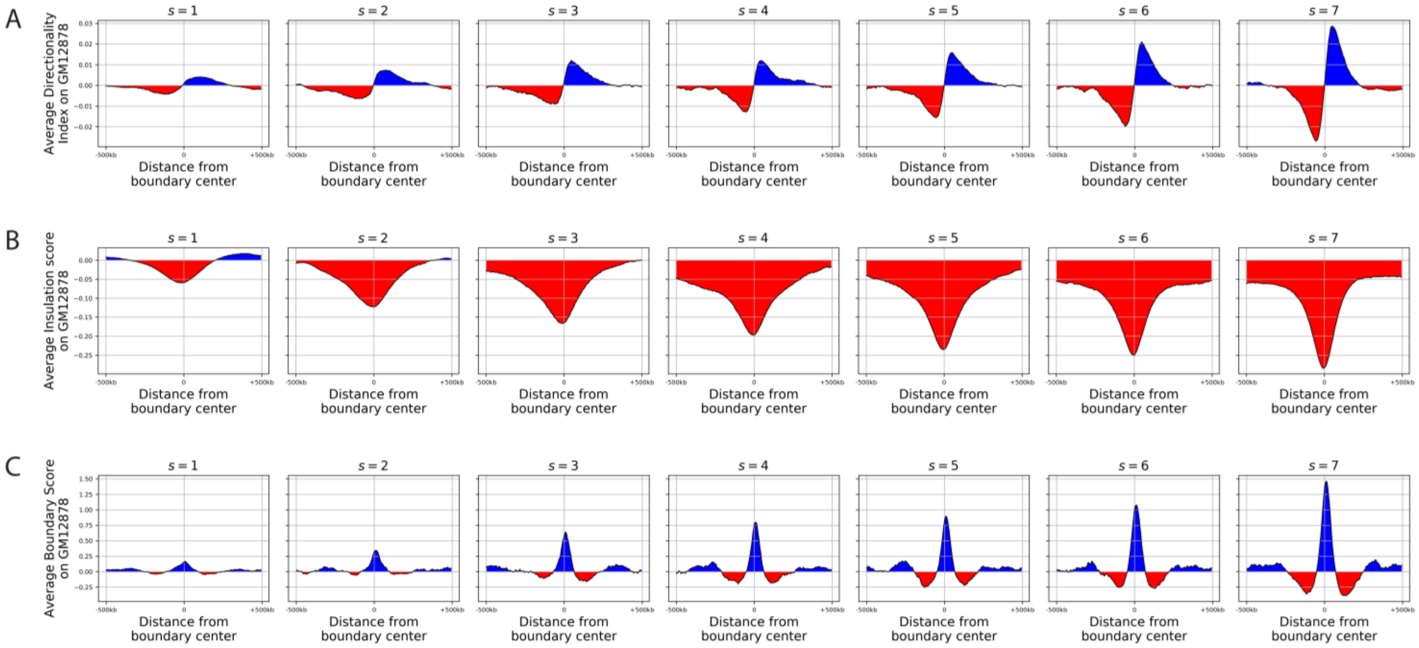


Fig. S6: The boundary identity gradient identified by the consensus algorithm provides a robust metric of boundary insulation

Average (**A**) directionality index [3], (**B**) insulation score [4] and (**C**) boundary score [5] in 5 kb windows, computed on GM12878 and projected onto the s1 to s7 boundaries in 1 Mb windows.

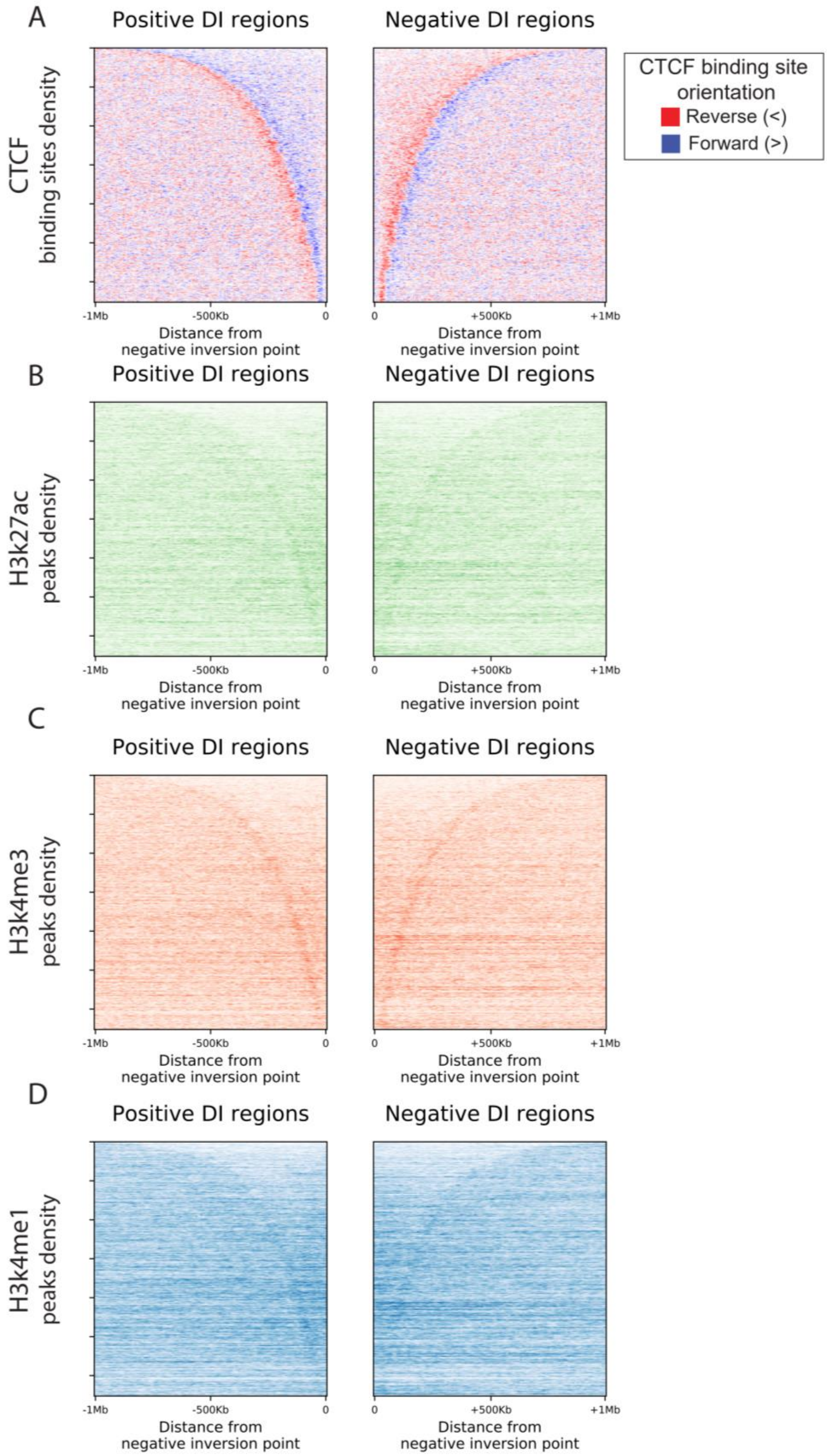


**Fig. S7**: Density of epigenetic marks in positive and negative directionality index TAD parts

(**A**) As Fig. 6G. (**B-D**) Density of H3K27ac (**B**), H3K4me3 (**C**) and H3K4me1 (**D**) on positive (*left*) and negative (*right*) directionality index regions on left and right hand topologically associated domain sections, ordered by size and aligned on their (+) to (-) DI sign inversion points.

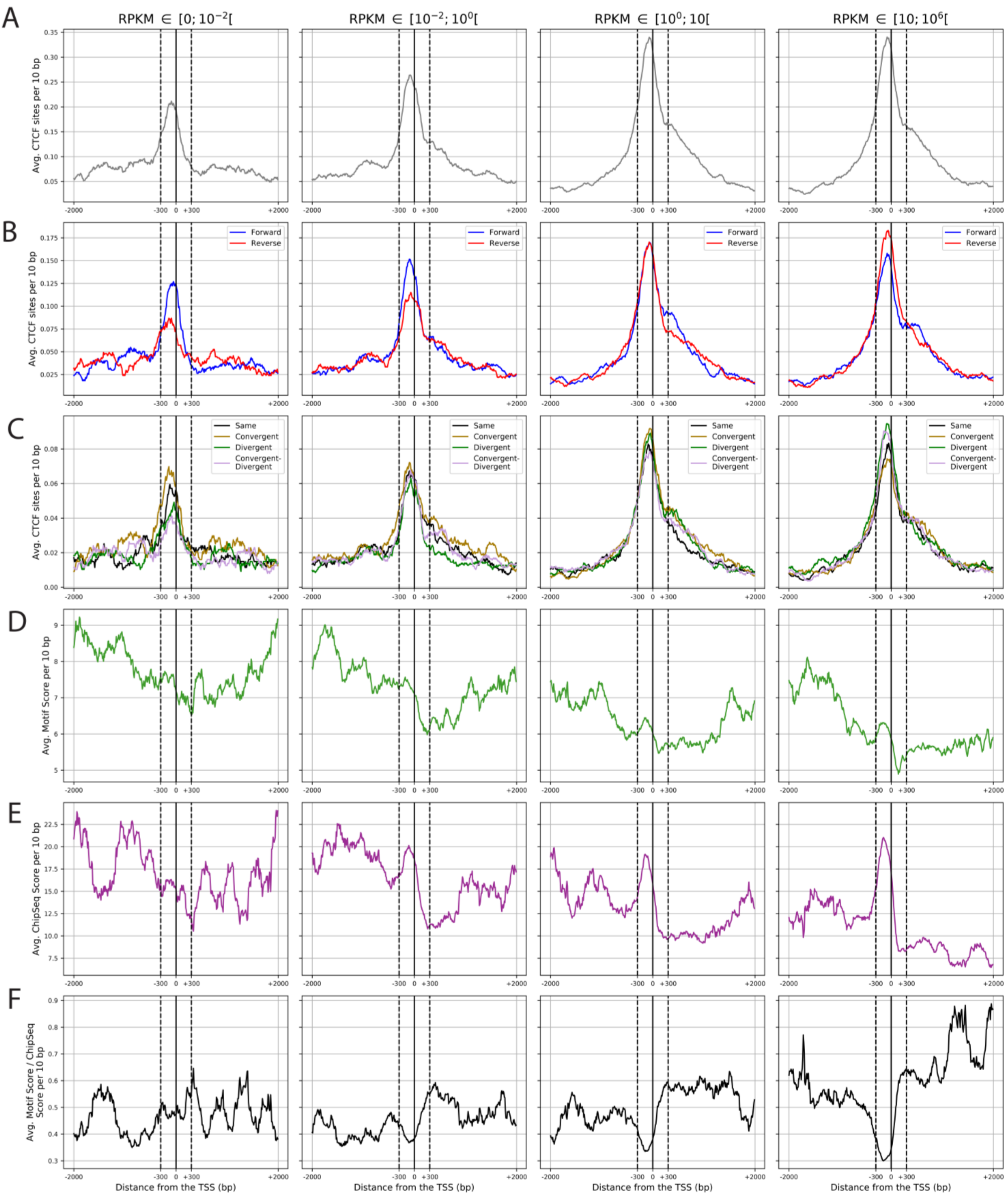


## Fig. S8: CTCF spatial classes do not correlate with gene orientation

Like **Fig. 1**, for each expression class of genes, (**A**) the density of CTCF binding sites (same as **Fig. 1A**), (**B**) the two CTCF site orientations, (**C**) the four CTCF orientation classes and (**D**) their motif score (same as **Fig. 1B**), (**E**) ChipSeq score (same as **Fig. 1C**) and (**F**) the ratio of motif and ChipSeq score (same as **Fig. 1D**). Plots are aligned on mRNA transcription start sites (TSS). and measures are averaged within 10 kb bins.

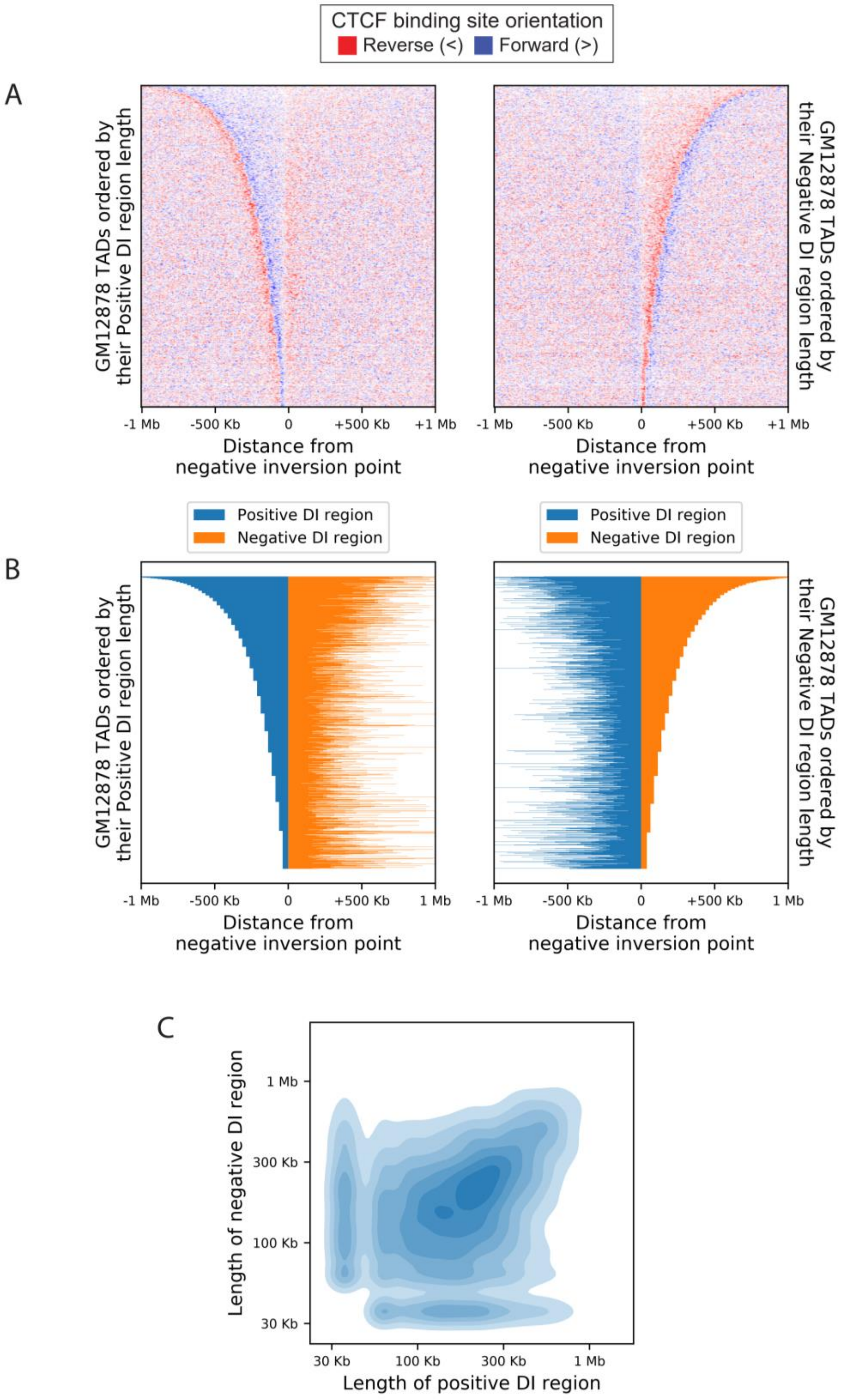


## Fig. S9: TADs can be divided in asymmetric halves by their directionality index negative inversion point

(**A**) Distribution of Forward (>, blue) and Reverse (<, red) CTCF sites in a 2 Mb window centred on the negative DI inversion points of 6,447 TADs extracted from GM12878. On the left the positive DI regions are ordered by their length from top to bottom and aligned with the negative inversion point to the right. On the right the negative DI regions are ordered by descending length and aligned with the negative inversion point to the left. Note that as the two TAD section sets are ordered separately, the regions at the same level in the two plots are not contiguous. (**B**) We show the effective size of the positive and negative DI regions for each of the 6,447 TADs from GM12878, ordered on the left by the size of positive DI regions, and on the left by the size of negative DI regions. (**C**) Density plot rendering the size of positive DI regions against the size of negative DI regions.

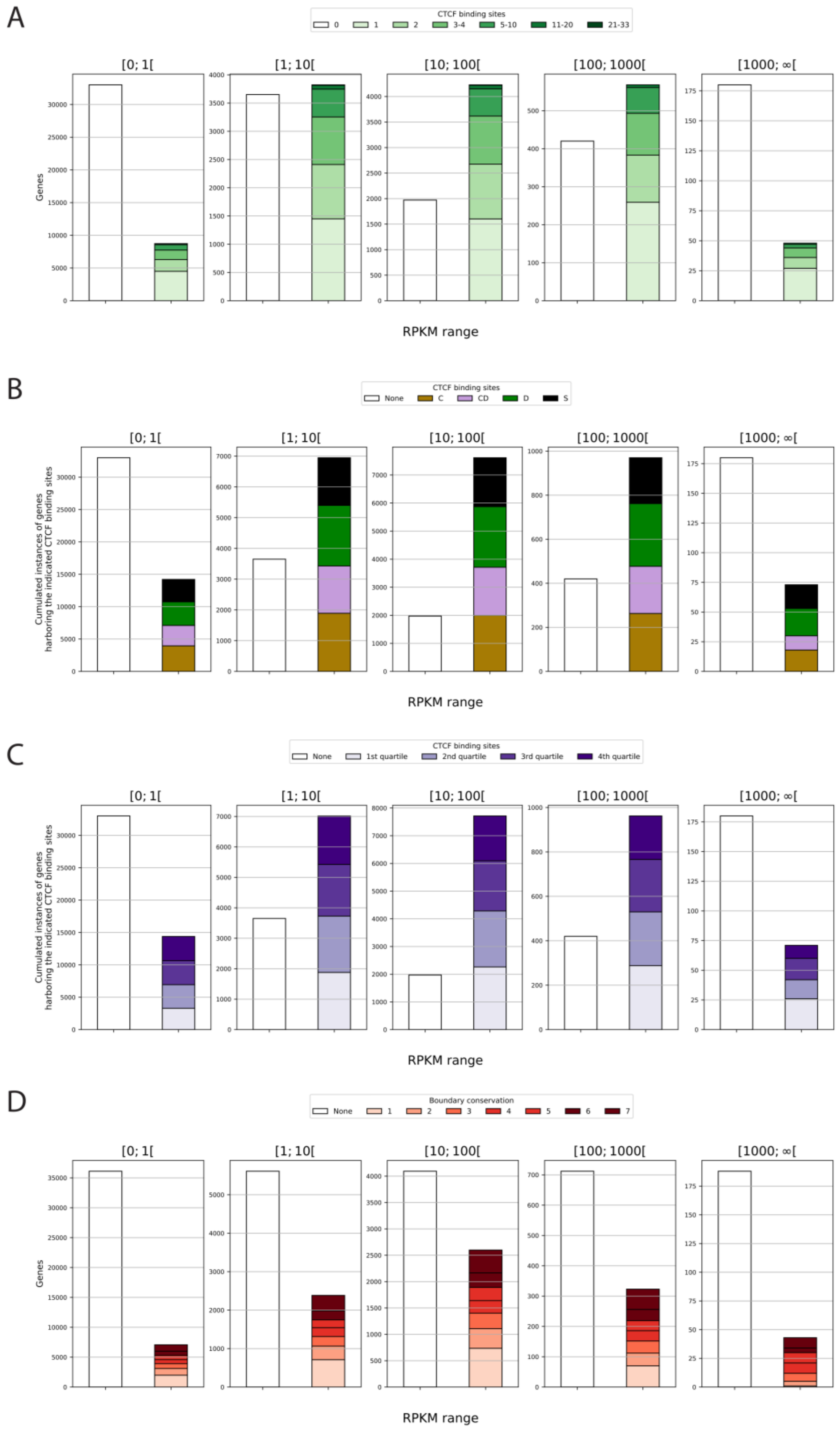


**Fig. S10**: Relationships between intragenic CTCF sites, TAD boundaries and gene expression

For the five indicated macrophage gene expression ranges extracted from Wang et al. 2019 [6] are shown: (**A**) the number of genes harbouring either no CTCF binding sites (white bar in all the panels) or one or more CTCF sites (green bars, with increasing intensity of green for larger counts), (**B**) instances of genes harboring the indicated CTCF binding site classes, (**C**) as (**B**) for CTCF binding site strength quartiles as defined by their rank aggregated score. (**D**) Number of genes intersecting either zero boundaries (white bar) or one to seven times conserved blood cell boundaries.

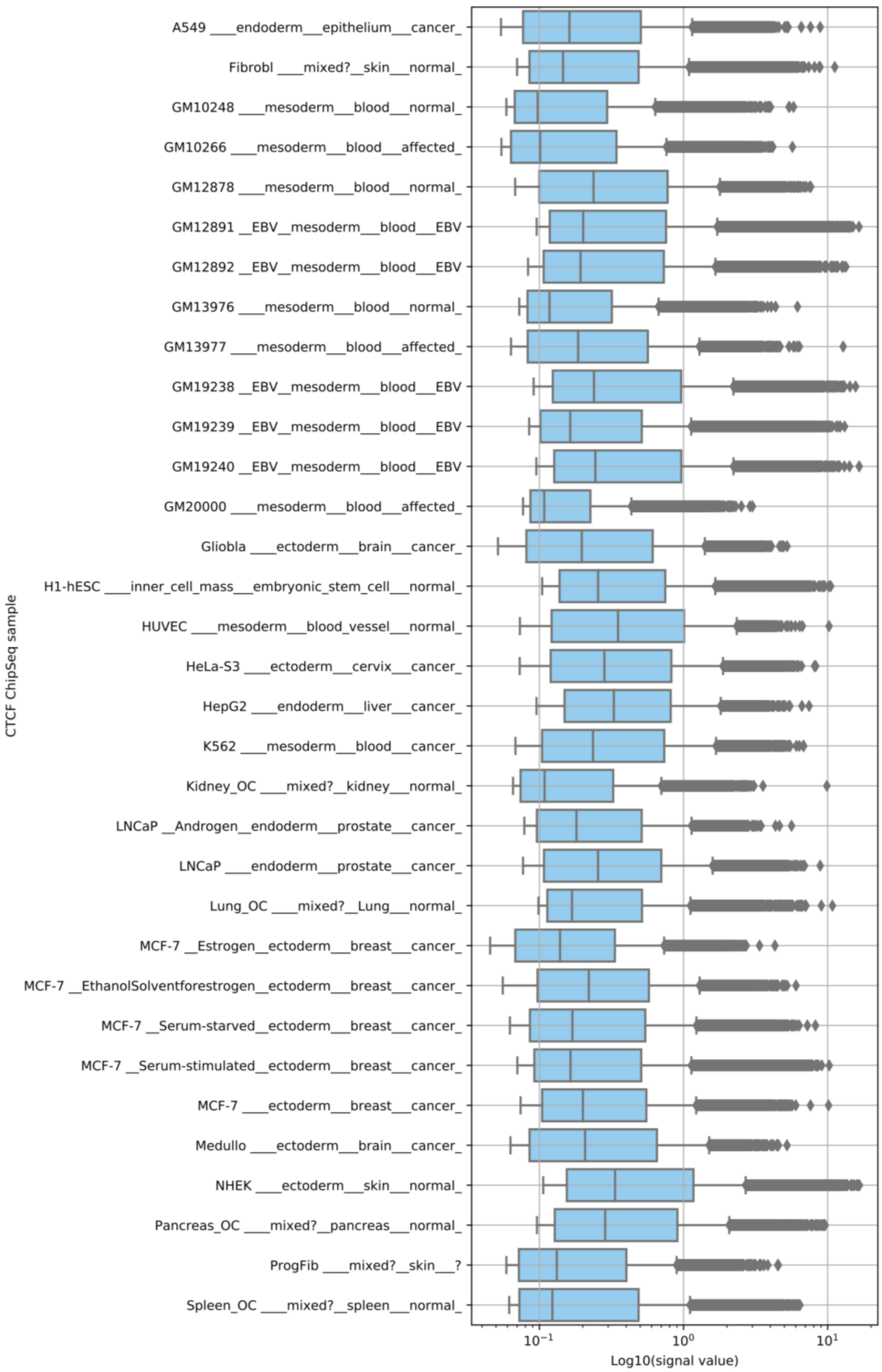


## Fig. S11: ChIPseq signal distribution of the 33 CTCF Narrow Peak tracks

For each ENCODE CTCF peak dataset, we show signal distribution on a log10 scale. The datasets come from various cell lines, laboratories and conditions (see **Table S2**). Therefore, the signal distributions are highly heterogeneous. In order to mitigate these biases, we performed a quantile normalization of each track with respect to the others (see Methods).

# References

1. Heinz S, Benner C, Spann N, Bertolino E, Lin YC, Laslo P, et al. Simple Combinations of Lineage-Determining Transcription Factors Prime cis-Regulatory Elements Required for Macrophage and B Cell Identities. Mol Cell. 2010;

2. Rao SSP, Huntley MH, Durand NC, Stamenova EK, Bochkov ID, Robinson JT, et al. A 3D map of the human genome at kilobase resolution reveals principles of chromatin looping. Cell. 2014;

3. Dixon JR, Selvaraj S, Yue F, Kim A, Li Y, Shen Y, et al. Topological domains in mammalian genomes identified by analysis of chromatin interactions. Nature. 2012;

4. Crane E, Bian Q, McCord RP, Lajoie BR, Wheeler BS, Ralston EJ, et al. Condensin-driven remodelling of X chromosome topology during dosage compensation. Nature. 2015;

5. Cresswell KG, Dozmorov MG. TADCompare: An R Package for Differential and Temporal Analysis of Topologically Associated Domains. Front Genet. Frontiers Media S.A.; 2020;11:158.

6. Wang C, Nanni L, Novakovic B, Megchelenbrink W, Kuznetsova T, Stunnenberg HG, et al. Extensive epigenomic integration of the glucocorticoid response in primary human monocytes and in vitro derived macrophages. Sci Rep. 2019;
