# Supplementary material for: Spatial patterns of CTCF sites define the anatomy of TADs and their boundaries
Source: Genome Biol. 2020 Aug 12;21:197. doi: 10.1186/s13059-020-02108-x (PMC7422557; doi:10.1186/s13059-020-02108-x)
Supplement: Supplementary file 5 — Additional file 5. Review history. [file 13059_2020_2108_MOESM5_ESM.docx]

Review History

**First round of review**

**Reviewer 1**

**Are you able to assess all statistics in the manuscript, including the appropriateness of statistical tests used?**

Yes, and I have assessed the statistics in my report.

**Comments to author:**

The authors characterize the relative orientations of CTCF sites within the human genome and correlate this with TAD organization. Although this is one of the first manuscripts I have seen to look at CTCF organization in combinations beyond simple pairwise relations, the overall findings are nothing new. Loops appear to be favoured between convergent orientations, so logically, TAD borders are more likely to have divergent CTCF sites, allowing for loops to be made with both the "left" and the "right" borders of adjacent TADs. I thus do not recommend publication in Genome Biology.

Other specific comments:

1. Quite a large part of the analysis depends on calling TADs and borders via the directionality index. This is particularly important in later analyses, where the authors analyse separately the "left" and "right" halves of TADs. This is not the only means of scoring borders, and in particular does not give a good metric of border "strength". Insulation scores (originally Crane et al., 2015, Nature, but subsequently used in many publications), or newer metrics which appear to call borders with even greater contrast (e.g. https://www.frontiersin.org/articles/10.3389/fgene.2020.00158/full) may be better suited.

2. While inherently a nice idea, I struggled to see how CTCF stratification helped with the downstream analyses, and so why it was really necessary. In any case, DNA methylation can be a major factor determining CTCF occupancy, which in turn could be a confounding feature (via unmethylated CpG islands at active genes) accounting for promoter biases in the results. Further, many CTCF sites are very far from genes; Figure 1A gives a very skewed view by only looking at the subset of CTCF sites that are promoter-proximal.

3. When comparing the number of "same" (16,025) vs "mixed" (14,378) CTCF triplets, is there a meaningful statistical test that can be done to show if this apparent slight bias is significant?

4. A large importance is placed to the results of Fig 3E, but I have two problems with this which I think downplays its significance. Firstly, it is not clear how many of the TAD borders actually qualify for the analysis - how many borders actually have 3 or more CTCF sites within the (sometimes) short distances requred in the analysis? For larger windows, is this even meaningful - are TAD borders actually that large, or is the analysis looking at multiple border combinations and/or "sub-border" close to the preferred borders? In other words, does the overall conclusion of convergent CTCF site enrichment at borders actually apply to the majority of borders? Secondly, the log10-scale presented in the figure implies that the enrichment is only <=1.14-fold anyway; a reliance of the conclusion on such a weak effect size should be justified.

**Reviewer 2**

**Are you able to assess all statistics in the manuscript, including the appropriateness of statistical tests used?**

Yes, and I have assessed the statistics in my report.

**Comments to author:**

This study is aiming at interpreting deeply linear DNA sequence information, i.e. CTCF binding sites, for the better comprehension of chromatin topological features, i.e. genome organisation into TADs. For their bioinformatics analysis, the author used a collection of approximately 60,000 human CTCF sites (described in Rao et al, Cell 2014). Interestingly, the authors define CTCF site orientation classes, resulting in well defined « patterns » of adjacent CTCF sites (including convergent and divergent site clusters), and analyze their linear spatial distribution along the genome. This work first show that, at relative short distance, i.e. 5kb to 100kb range, divergent CTCF site clusters are enriched, while convergent CTCF clusters are depleted. These regions enriched in divergent CTCF site patterns correspond to highly conserved TAD boundaries. Second, at negative inversion sites of chromatin interactions inside TADs, the opposite situation was found, indicating that the anatomy of CTCF sites may reveal specific genome organization features. Therefore, an alternation of divergent and convergent CTCF site clusters seem to exist at different scale lengths along the genome, which may be important for its organization into TADs, sub-TADs and boundaries. Overall, this bioinformatic study is rather informative for the field, clear to follow and well presented. I therefore recommend its publication in Genome biology, with the following minor revisions.

- Figure 2: for mono-plets, would have make sens to also indicate the number of forward and reverse pointing. Here, only the total number of CTCF sites is indicated (61,079).

- How it is possible that the Convergent and Divergent triplet CTCF sites has exactly the same number of occurence (15,315, Fig.2). The authors should comment on that. Also, I would have expected the number of possible occurence for each patterns to be equal to the total number of CTCF site analyzed (61,079). Is there a trivial explanation here?

- In the Table of Fig.2, Mention « patterns » in the table on Top of the column containing « Same, Convergent, Divergent, Conv-Div ».

This would avoid confusion in the text with « the number of possible permutations ».

- When the authors mention an evolutionary gain of same « orientated triplets », is it a way to measure this statistically when compare with convergent, Divergent and Conv-Div.

- Fig.3B: randomization is performed, indicating that the « real » distribution is biased. The authors should calculate a statistical score associated with this analysis. Same remark for Figure 3C.

- line 184: the authors claimed that "Convergent di-plets are depleted when using the entire CTCF set (Fig.3E), but become enriched when the top quartile CTCF set is considered (Fig S2C)". However, this doesn't seem to be the case or may require some clarification.

- line 244: I was not sure how the authors calculate that 16% of S1-S7 boundaries lack a CTCF site. It is actually an important point to mention. Actually, it would have been nice to have the percentage for each « S » category in order to appreciate that the highly conserved boundaries are the most enriched in CTCF binding sites.

- Concerning epigenetic marks at TAD boundaries, the authors report a « weak but discernible enrichment of H3K27ac and H3K4me1 marks around some negative DI inversion sites ». Those marks often correspond to enhancers. Actually a clearer enrichment of H3K4me3, the mark of TSS/promoters is also observed at this negative inversion sites inside TADs, which would indicate that gene promoters and enhancers could have a biais position inside TADs. Something to clarify/discuss in the text.

- For the discussion, their representation of TAD as <>(>…>)(<…<)<> with divergent CTCF sites at TAD boundaries and convergent CTCF sites inside TAD halves is clearly a novelty for the field. However, it is a pity that the authors doesn't take advantage of this finding to extend a bit their discussion toward an evolutionary perspective (see for example, Vietri Rudan et al, Cell Rep 2015 paper to extend the discussion part).

**Authors Response**

**Point-by-point responses to the reviewers’ comments:**

Reviewer 1, general comment:

The authors characterize the relative orientations of CTCF sites within the human genome and correlate this with TAD organization. Although this is one of the first manuscripts I have seen to look at CTCF organization in combinations beyond simple pairwise relations, the overall findings are nothing new. Loops appear to be favoured between convergent orientations, so logically, TAD borders are more likely to have divergent CTCF sites, allowing for loops to be made with both the "left" and the "right" borders of adjacent TADs. I thus do not recommend publication in Genome Biology.

*We are glad to see that the reviewer appreciates that our manuscript is novel in its characterization of combinations of CTCF sites beyond pairs of sites. The reviewer is correct when s.he states that loops appear to be favored between convergent orientations of pairs of CTCF sites, and that this is well-known. But, we do not believe a convincing demonstration, beyond the ‘thought experiment’, that TAD borders are more likely to have divergent CTCF sites, allowing for loops to be made with both the "left" and the "right" borders of adjacent TADs’ had been made yet. This is because the formalism had to extend beyond pairs of CTCF sites! Indeed, prior to our work it was not known how CTCF relative orientations define the TAD level and the boundary level. Rather, much of the current literature points either to CTCF site density to explain TAD-level features, or to gene regulation via enhancer-promoter contacts that are facilitated by convergent CTCF sites. As a matter of fact, it could have been argued that a convergent CTCF pair would be the strongest border itself, as it would be insurmountable by the extrusion system if the CTCF sites were to halt every extrusion complex. That is not the result we obtained! Hence, our work is novel in that it identifies a CTCF grammar that defines TAD boundaries and TAD interiors, independently of the role of promoter-enhancer looping via convergent CTCF sites or the identification of CTCF-dense regions as more likely TAD borders.*

*Below we set out a line of argumentation, from general to most specific, to defend the novelty of our work.*

*First and foremost, we wish to point out that in general loops and TADs are not completely overlapping, because the former are the “building blocks” of the latter. TAD features should be considered as a type of continuant while loops are a type of occurant. In other words, in any one cell, only one loop (extrusion) conformation can exist at any one point in time, while TAD-level features represent the outcome of the observation of a near infinity of cellular chromosome loop combinations. However, the novelty of our work does not lie in the realization that chromatin loop extrusion shapes TADs and their boundaries. Rather, it is the enunciation of a CTCF grammar that considers CTCF sites combinations of more than two CTCF sites that is novel. In our opinion, the fact that the rules are elegant and seem, a-posteriori, to be obvious does not take away from the fact that we are the first to have formalized them and that we are the first to have devised bioinformatic approaches to unravel and visually display the results of these investigations. Indeed, we are still fascinated by the aesthetics of panels 6G and S8A of the revised manuscript, which, to our knowledge have not been reported by anyone to date.*

Reviewer 1, specific comments:

1. Quite a large part of the analysis depends on calling TADs and borders via the directionality index. This is particularly important in later analyses, where the authors analyse separately the "left" and "right" halves of TADs. This is not the only means of scoring borders, and in particular, does not give a good metric of border "strength". Insulation scores (originally Crane et al., 2015, Nature, but subsequently used in many publications), or newer metrics which appear to call borders with even greater contrast (e.g. https://www.frontiersin.org/articles/10.3389/fgene.2020.00158/full) may be better suited.

*In our work, we used a collection of TAD boundaries in seven primary human types from the Blueprint Consortium (Javierre et al. 2016) as they were calculated by the original authors using the Directionality Index method (Dixon et al. 2012). One of our deliverables is a novel consensus algorithm that provides a gradient of boundary identity, which we propose is a proxy for boundary strength. As described in the Methods section of the paper, the algorithm aggregates boundaries from different cell types. Since our findings are based on this somewhat coarse consensus, we believe that increasing the specificity of boundary calling would not yield significantly different results. Instead, in the second part of the work we calculate boundaries from the GM12878 cell line from Rao et al. 2014 [3]. In our original manuscript we used the Directionality index for coherence with the method used in Javierre et al.*

*We provide below a novel comparison using two boundary strength scoring methods, so as to clarify the relationships between the gradient of 7 levels of boundary identity we had generated and other means of determining TAD boundary strength. The analysis shown originally in Figure 4G presented a linear increase of the sharpness of DI index sign change at boundaries of increasing conservation score. To address Reviewer 1, we performed analyses on GM12878 HiC data, which is publicly available [3], using the Insulation score [4] as well as the boundary score calculated from the TADCompare package suggested by the reviewer [1]. We are glad to report that these two new analyses, shown on Rebuttal Figures 1 and 2, and now included in the revised manuscript as supplementary Figure S5, yielded very similar results. This confirms the validity of our approach using the boundary identity gradient to identify chromosome folding grammatical elements made up of CTCF triplet combinations.*

*Rebuttal Figure 1: Plot of distance to boundary center against insulation score (log2) [4] as calculated by the cooltools package [5].*

*Rebuttal Figure 2: Plot of distance to boundary center against the ‘Boundary Score’ as calculated by the TADCompare package [1].*

*Altogether, we believe these two new analyses satisfy the proposition that our boundary consensus algorithm and the resulting boundary identity gradient we exploited in the paper do give a good metric of ‘border strength’.*

2. While inherently a nice idea, I struggled to see how CTCF stratification helped with the downstream analyses, and so why it was really necessary. In any case, DNA methylation can be a major factor determining CTCF occupancy, which in turn could be a confounding feature (via unmethylated CpG islands at active genes) accounting for promoter biases in the results. Further, many CTCF sit es are very far from genes; Figure 1A gives a very skewed view by only looking at the subset of CTCF sites that are promoter-proximal.

*We thank the reviewer for finding it a nice idea to stratify CTCF sites. Furthermore, we invested some effort in the investigation of possible biases due to DNA methylation of CTCF sites at promoters.*

*First, we wish to stress that except for Figures 1, S2, S7, none of our CTCF pattern analyses focused on gene promoters. Instead, they were performed genome-wide and in an unbiased fashion, considering all CTCF sites and their orientation (Figures 2, 3, 4, 5, 6, S1, S3, S4, S5, S6, S8, S10), or considering genes from their start till their end in the context of expression strength (Figure S9).*

*Second, to put the results shown in figure 1 in a genome-wide context, we plot below the extent of CTCF occupancy bias and CTCF signal and motif strength in promotor regions. Of the 61,079 CTCF sites that we studied, 8,846 reside in 7,747 protein-coding gene promoters equipped with at least one CTCF site, when such are defined as 4 kb regions around the gene’s TSS (Rebuttal Figure 3). This demonstrates a very strong enrichment of CTCF occupancy at promoters (Fisher exact test for overlapping p-value = 0, as calculated by the bedtools software), and that is why in the manuscript we focus on promoters in figures 1 and S7, and on genes in figure S9. Indeed, and importantly, Rebuttal Figure 3 indicates that the majority of promoter-CTCF sites inhabit promoters of moderately to strongly expressed genes (10-106 RPKM), which is also shown in the manuscript in figure S2C).*

*Rebuttal Figure 3: CTCF site prevalence at promoters as a function of gene expression strength.*

*However, as explained succinctly in the manuscript and as visible in Figure 1, we noticed that high CTCF occupancy does not directly imply the presence of a strong binding motif instance. To check whether any CTCF ChIP signal strength biases are present as a function of genomic location, and in particular gene promoters, which are thought to be important for gene promoter-enhancer contacts (see also the response to general comment Reviewer 1), we analyzed CTCF ChipSeq signals and motif scores inside and outside promoter regions elaborately and thoroughly.*

*This analysis is described in the materials and methods section (see also Figure S7) and gave rise to Figure 1 which documents clear instances of the disconnect between CTCF ChIPseq signal and CTCF Motif strength as one travels from 300 bp upstreaam the TSS to 300 bp downstream (another finding that is new, to the best of our knowledge). That disconnect inspired us to combine the two CTCF binding site metrics into an aggregate rank score that we went to validate as a superior CTCF site ‘strength’ measure at the hand of loop extrusion simulations outcomes and HiCCUPS, applying stringent standards of recall and precision measures (Figure S1E-F).*

*The CTCF site strength stratification served to investigate CTCF site ‘strength’ effects in revised manuscript figures S2, S7 and S9C. Below, in Rebuttal figures 6 we again employ our independently validated (Figure S1E-F) novel CTCF-site stratification approach to address reviewer 1’s comment on the potentially confounding effect of DNA methylation on our promoter CTCF binding analysis. Globally, Rebuttal Figures 4, 5 and 6, indicate that promoter CTCF sites do not dominate the system as they are not significantly stronger than non-promoter CTCF sites, quite the contrary. We thank the critical question of reviewer 1 for it led us to revise figures 1B and 1C, and to include a new supplementary figure (revised manuscript Fig. S2) so as to demonstrate that point explicitly.*

*Rebuttal Figure 4: t-test based statistical analyses of CTCF site strength outside and inside gene promoters.*

*Rebuttal Figure 5: CTCF site strength aggregate rank score distributions plotted as a function of gene promoter strength stratified as in figure 1 of the manuscript.*

*Rebuttal Figure 6: Number of CTCF sites with weakest (quartile 1) to strongest strength (quartile 4), plotted inside and outside gen promoters.*

*Figure 1 of the manuscript gives a vision of CTCF enrichment around the TSS of genes, and therefore does not provide insight as to the CTCF sites along the rest of the genome. This means that the enrichment/depletion between close-to-TSS and far-from-TSS binding sites is relative. Once we undertake a whole-genome analysis, then we can see that the signal intensities are not biased towards promoter regions (Rebuttal Figure 4). Notably, this validates our decision to indiscriminately study CTCF sites inside and outside promoters in Figures 2, 3, 4, 5, 6, S1, S3, S4, S5, S6, S8, S10. Indeed, it is noticeable that CTCF site strength metrics are statistically weaker in promoter regions, relative to the rest of the genome (Rebuttal Figures 5 and 6). Hence, promoter-enhancer looping via convergent CTCF sites involving promoter CTCF sites are unlikely to dominate the global chromosome looping landscape, neither by numbers and nor by strength, although in individual instances they certainly do.*

*When we performed the sensitivity analysis (Figure S4 of the revised manuscript) on our novel genome-wide CTCF orientation pattern analysis we observed that the biases are conserved even when retaining only top-ranked CTCF binding sites, revealing quite a stable overall configuration of CTCF orientations. In light of these new analyses, we can confidently state that our genome-wide findings on spatial distribution biases of relative CTCF orientation classes are not strongly affected by promoter-level biases.*

*Below, we carefully address the second part of Reviewer 1’s second comment, namely the potentially confounding influence of DNA methylation on the results displayed on Figure 1 of the manuscript.*

*Rebuttal Figure 7: Left: beta-distribution of the GM12878 cell DNA methylation data (Myers, RRBS - https://www.encodeproject.org/experiments/ENCSR000DEY/). Middle: DNA methylation data calls for CTCF motif instances outside and inside gene promoters. Right: DNA methylation beta-value distribution for CTCF sites inside and outside gene promoters.*

*Rebuttal Figure 8: Spatial distribution of DNA methylation data around transcription start sites of human protein-coding genes (independently of CTCF sites) plotted as a function of gene expression strength.*

*Rebuttal Figure 9: Spatial distribution of CTCF motifs located around the transcription start sites of human protein-coding genes that do and that do not have DNA methylation data, plotted as a function of gene expression strength.*

*Rebuttal Figure 10: Spatial distribution of CTCF motifs located around the transcription start sites of human protein-coding genes with high and low and with DNA methylation levels, plotted as a function of gene expression strength.*

*CTCF-DNA interaction is methylation sensitive on C(2) and C(12) of the sequence GCCAGCAGGGGGCGCTA. These two cytosines in the consensus sequence can be methylated and have opposite effects on CTCF-DNA interactions, suggesting that “CTCF-binding sites may exist in the genome in four different methylation states with varied affinities for CTCF” [6]. The RRBS DNA methylation data set for GM12878 that we employed to answer the reviewers concerns about CTCF site methylation is strongly biased towards promoters (Rebuttal Figure 7 middle panel, reference data set), whereby 4,764 of the 8,846 promoter CTCF sites residing in the vicinity of the promoter transcription start sites (TSS) we analyzed do have DNA methylation data. Fortunately, this does enable the analysis of promoter CTCF site methylation in relation to promoter strength (Rebuttal Figures 7 and 8). The subset of this methylation data that concerns CTCF motifs within CTCF bound regions displays a very similar beta distribution as the entire data set. Therefore, we do not expect a confounding effect at the level of CTCF motifs methylation representation in the methylation data set (Rebuttal Figure 7). Furthermore, CTCF sites with available DNA methylation data display very similar distributions around the TSS as the entire population that was plotted on manuscript Figures 1A-D, arguing that there will also not be a confounding issue at the level of spatial distribution of the CTCF sites with methylation data around the TSSs (Rebuttal Figure 9). Finally, stratifying the CTCF ChIPseq sites by the methylation status of the underlying CTCF motifs revealed that 612 bear a methylated cytosine, while 4170 do not (Rebuttal Figure 10, left versus right panels). Notably, the spatial distribution of the ratios of CTCF ChIPseq-signal to Motif strength is highly similar to the original manuscript Figure 1D (Rebuttal Figure 10, bottom right panel).*

*Altogether, the above analyses demonstrate that the low methylation CTCF site subset is representative for the entire CTCF population. The large majority of promoter CTCF sites are not methylated. Crucially, the unmethylated CTCF binding sites still display the 50% increase in ChIPseq to Motif score ratio when comparing CTCF sites that lie less than 300 upstream of the TSS to those that lie less than 300 downstream of it, when highly expressed genes are considered (Rebuttal Figure 10, bottom panels). We therefore conclude that the potential concern of Reviewer 1 as to the potentially confounding effect of CTCF site cytosine methylation is not supported by the DNA methylation data, since there is very little DNA methylation of the promoter CTCF motifs we study in Figure 1. As all these are negative conclusions, we do not wish to include the DNA methylation analysis in the revised manuscript.*

3. When comparing the number of "same" (16,025) vs "mixed" (14,378) CTCF triplets, is there a meaningful statistical test that can be done to show if this apparent slight bias is significant?

*We thank the reviewer for suggesting to strengthen this aspect of our analysis. Indeed, there are several possible tests and all of them show that the “bias” is very significant.*

*We first performed a classic Pearson Chi-Squared test for the values of Figure 2, for the bi-, tri- and tetra-plet patterns. In particular, we tested whether the number of patterns found for each length (2, 3 and 4) are unbalanced with respect to a uniform distribution. For example, in the case of tetra-plets we have 2 same, 3 convergent, 3 divergent and 8 convergent-divergent possible patterns. This means that in a uniform distribution we expect 12.5% of patterns to be same, 18.7% to be convergent, 18.7% to be divergent and 50% to be convergent-divergent. Given our expectations, we can then calculate the Chi-square statistic for tetra-plets. We applied the same reasoning for the other pattern lengths.*

*We obtained the following values:*

*●	Biplets:	pvalue = 1.95e-10	stat = 44.712*

*●	Triplets:	pvalue = 2.337e-19	stat = 89.86*

*●	Tetraplets:	pvalue = 1.37e-33	stat = 155.94*

*As expected, the significance increases when longer patterns are considered, since the number of possibilities increases and the bias becomes more prominent. These significances are now reported in revised Figure 2.*

*We then considered a more data-driven approach, based on the randomization of the CTCF site orientations. For bi-plets, tri-plets and tetra-plets we performed the following steps:*

*1.	Calculate the theoretical expected value for the proportions of classes (for example, in the case of triplets will be Same = 0.25, Conv = 0.25, Div = 0.25 and Conv. Div = 0.25; in the case of biplets instead we have Same = 0.5, Conv = 0.25, Div = 0.25; and so on...)*

*2.	Permute the orientations of CTCF sites in the genome (keeping their position fixed)*

*3.	Calculate the frequencies of each pattern category, building, therefore, an empirical expected value, one for each randomization; we used 10,000 randomizations.*

*4.	Calculate a distance measure between the frequency values of each randomized expected and the theoretical expected values.*

*5.	Build a distance distribution; we used the Chi-squared statistic*

*∈{,,,}*

*6.	Finally, we compare the distance between the observed frequencies and the theoretical ones with the previously computed distribution.*

*In this way, we have a visual and quantitative assessment of the shift of our data, divided by the size of the pattern. All the skews (which can be calculated as empirical p-values) are very significant and they all converge to zero. Rebuttal Figure 11 has been added to the manuscript as Supplementary Figure S3. A brief explanation of this randomization test has been added to the methods part of the paper.*

*Rebuttal Figure 11: Randomization-based statistical analysis of the bias in representation of bi-plet, tri-plet and tetra-plet CTCF patterns.*

*We also wish to point reviewer 1 to our response to comment 2 of reviewer 2, which led us to fix the numbers reported in Figure 2 by splitting chromosomes at the centromeres.*

4. A large importance is placed on the results of Fig 3E, but I have two problems with this which I think downplays its significance. Firstly, it is not clear how many of the TAD borders actually qualify for the analysis - how many borders actually have 3 or more CTCF sites within the (sometimes) short distances required in the analysis? For larger windows, is this even meaningful - are TAD borders actually that large, or is the analysis looking at multiple border combinations and/or "sub-border" close to the preferred borders? In other words, does the overall conclusion of convergent CTCF site enrichment at borders actually apply to the majority of borders? Secondly, the log10-scale presented in the figure implies that the enrichment is only <=1.14-fold anyway; reliance on the conclusion on such a weak effect size should be justified.

*We thank Reviewer 1 for pointing out the need for a better statistical validation of the results presented in Figure 3E as this has led to an improved version of manuscript original Figures 3E (3F in the revised manuscript) and Supplementary Figure 2 (revised manuscript S4) and the generation of a new Figure 3 panel, (Revised manuscript panel 3E).*

*First of all, it is important to clarify that the entire analysis shown in Figure 3 was performed genome-wide and unbiasedly, with the major objective of understanding which, if any, CTCF pattern classes (Convergent, Divergent, Convergent-Divergent and Same) have unexpected spatial distribution. It has therefore not been performed using any known or inferred TAD borders. With ‘spatial distribution’ we specifically mean at which distance “regime” (which we term “clustering window” in the text and figures) the CTCF sites belonging to the studied patterns operate. The question comes from the hypothesis that topological features like TADs and their boundaries, which are distributed along the genome at different length scales (with TADs usually spanning 100 kb to 1 Mb, while boundaries are more in the 1-50 kb range), should be encoded by arrays/configurations of CTCF sites having similar spatial span. In Figure 5A we do show, for each boundary conservation score, how many boundaries have 0, 1, or more CTCF binding sites around their center ± 25 kb.*

*In order to provide statistical treatment, in Figure 3F we had shown the enrichment/depletion only of those patterns whose inter-CTCF distance is less or equal than the indicated windows. We compared the found counts against a uniform distribution, one for each pattern length. Our hypothesis was that patterns encoding boundaries and TADs would be enriched at windows compatible with the length distribution of these topological features. In the revised Figure 3F (Rebuttal Figure 12), clustering windows grow in log2 scale. Because there was no difference for window sizes greater than 1Mb the figure includes window sizes only up to 2Mb. This is because after that length threshold, virtually all CTCF sites are considered in the analysis and the numbers converge to the complete set shown in revised Figure 2.*

*Rebuttal Figure 12: Replacement for original manuscript Figure 3E (now Figure 3F) where the window sizes are appropriate and increase logarithmically.*

*In the original version of figure 2, we had constrained the log10(enrichment) color scale between -0.06 and +0.06 (also shown in the new version of the figure). This was done to better highlight the enrichment of patterns also in window regions where the skew from random was less prominent than the maximum value found (see Rebuttal Figure 14). For completeness, on Rebuttal Figure 13 we also display the enrichment map colored according to a constraint between -0.11 and +0.11 (equivalent to a 1,28-fold change), corresponding to the maximum enrichment/depletion value found in the analysis. This clearly shows that the divergent patterns are enriched between 8 and 38 kb, indicating enrichment for the smallest feature of TADs, which are likely the TAD boundaries, although this analysis cannot demonstrate this, as it does not include knowledge of boundary identity or any HiC data, for that matter. The crux of the qualitative argument, however, is that divergent and convergent CTCF sites must have the same cardinality because mathematically, they are obligate alternating features of CTCF patterns (!). Hence, any divergence in their length scale distributions cannot be caused by local differences in the abundance of these patterns in supra-megabase domains, since they must systematically alternate, as explained in the manuscript in the results, discussion and methods sections (see also our response to comment 2 of reviewer 2).*

*Rebuttal Figure 13: Version of replacement for manuscript Figure 3E where the color scales cover 1.3 fold, instead of 1.14-fold.*

*Finally, to quantitatively evaluate the statistical significance of our findings we adopted the same strategy described for the whole-genome CTCF pattern count analysis in response to Reviewer 1 comment 3, above. Specifically, for each clustering window w, we compute the number of Same, Convergent, Divergent and Convergent-Divergent patterns. We then calculate the test statistics and p-values from a Pearson Chi-Square test, for each window w, checking if the class proportions are significantly skewed. P-values converge to those of our response to point 3 at the window size of 1Mb, since at this clustering window level a single big cluster is formed for each chromosome arm, but they are dramatically lower at window sizes between 8 kb and 33 kb (Rebuttal Figure 14), highlighting strong biases at these windows. As expected, the values at very small windows are not significant, since the probability of having two binding sites less than 1 kb is low. This plot of negative log10 p-values of the Pearson Chi-Squared test as a function of the window size is now shown as a new panel in the revised manuscript as Figure 3E.*

*Rebuttal Figure 14: Visualization of the statistical validation for Figure 3E, correcting for multiple testing using Bonferroni-corrected P-values obtained using the Pearson chi-square test, for skews in the representation of bi-plets, tri-plets and tetra-plets as a function of genomic window size and plotted on a log2 (bp)scale. This figure is the new panel E of revised manuscript Figure 3.*

*As the P-values displayed on Rebuttal Figure 14 measure the unbalance of the configurations but do not give an explicit account for the distribution of same, convergent, divergent and convergent-divergent patterns, we still need revised manuscript Figure 3F to understand which classes are enriched/depleted at the various windows.*

*The results of revised manuscript Figure 3F were subjected to a sensitivity analysis (revised manuscript supplementary Figure 4) and the biases identified using the complete set of CTCF sites are indeed still retained when removing low-scoring binding sites, showing an embedded configuration at the genome-wide level. We have redrawn Supplementary Figure 2 with the new window scale, and this can be seen also below in Rebuttal Figure 15.*

*Rebuttal Figure 15: Sensitivity analysis using the new log2 distance scale employed to improve Figure 3E. This is the new version of the original figure S2 (now Figure S4).*

*To better highlight the behavior of the CTCF patterns in “boundary-range” windows between 8 and 65 kb, we repeated the count of these patterns within bi-plets, tri-plets and tetra-plets, only for the patterns whose elements are no more than 25 Kb apart. These results are shown on Rebuttal Figure 16. Comparison with the counts for full-genome, reported in Manuscript Figure 2, shows very skewed counts indeed, with a noticeable enrichment of divergent patterns for every pattern length.*

*Rebuttal Figure 16: Real numbers of CTCF pattern clusters in windows of 25 kb or less underlying the peaks in statistical significance displayed on Rebuttal Figure 14.*

*This is best exemplified at the level of 3-plets. Theoretically, in a genome-folding system in which the only function of CTCF binding sites is to regulate loop extrusion, the only possible pattern configuration is a regular sequence of Same/Conv.Div. - Convergent – Same/Conv.Div. - Divergent – Same/Conv.Div. - Convergent ...., which would in turn produce an equal number of Convergent and Divergent patterns. The balancing of Convergent and Divergent patterns does indeed happen when we look at the full-genome configurations (Manuscript Figure 2). However, below 25 kb, while Convergent-Divergent patterns are present but only slightly depleted due to the real sequence of binding site orientation, the counts are shifted quite dramatically in favor of Divergent and Same sites, which are present 5,320 and 5,482 times, respectively, while convergent patterns only occur 4,362 times (Rebuttal Figure 16). Rebuttal Figure 14 indicates that the probability of observing such depletion at 25 kb is of the order of 10-33 as calculated with the Bonferroni-corrected Pearson chi-square statistical test.*

Reviewer 2

This study is aiming at interpreting deeply linear DNA sequence information, i.e. CTCF binding sites, for the better comprehension of chromatin topological features, i.e. genome organisation into TADs. For their bioinformatics analysis, the author used a collection of approximately 60,000 human CTCF sites (described in Rao et al, Cell 2014). Interestingly, the authors define CTCF site orientation classes, resulting in well defined « patterns » of adjacent CTCF sites (including convergent and divergent site clusters), and analyze their linear spatial distribution along the genome. This work first shows that, at relative short distance, i.e. 5kb to 100kb range, divergent CTCF site clusters are enriched, while convergent CTCF clusters are depleted. These regions enriched in divergent CTCF site patterns correspond to highly conserved TAD boundaries. Second, at negative inversion sites of chromatin interactions inside TADs, the opposite situation was found, indicating that the anatomy of CTCF sites may reveal specific genome organization features. Therefore, an alternation of divergent and convergent CTCF site clusters seem to exist at different scale lengths along the genome, which may be important for its organization into TADs, sub-TADs and boundaries. Overall, this bioinformatic study is rather informative for the field, clear to follow and well presented. I therefore recommend its publication in Genome biology, with the following minor revisions.

*We are grateful to the reviewer for recognizing the novelty of our work, for summarizing our results so nicely and for recommending acceptance. Below, we respond to the requested minor revisions.*

R2.1 Figure 2: for mono-plets, would have made sense to also indicate the number of forward and reverse pointing. Here, only the total number of CTCF sites is indicated (61,079).

*This information has been added to Figure 2. See also R2.2 response.*

R2.2 How it is possible that the Convergent and Divergent triplet CTCF sites has exactly the same number of occurrences (15,315, Fig.2). The authors should comment on that. Also, I would have expected the number of possible occurrences for each pattern to be equal to the total number of CTCF site analyzed (61,079). Is there a trivial explanation here?

*First of all, we thank the reviewer for this question, since it enabled us to spot a tiny inaccuracy in our pattern count calculation code for Manuscript Figure 2, leading to a slight change of the reported numbers, not affecting any of the downstream results of our analysis in any case. The new figure has been updated and is also shown in Rebuttal Figure 17.*

*Rebuttal Figure 17: Updated Manuscript Figure 2*

*Indeed, in the new numeration the number of convergent and divergent tri-plets is not identical but has a difference of 1. We now explain in theRresults and Methods sections how the Convergent and Divergent tri-plets are built, which makes their number of occurrences mandatorily identical with a maximal difference of 2 counts per linear DNA stretch. We also refer the reviewer to the Methods section “Properties of the CTCF subsequences”. Below, we explain briefly how the numeration works.*

*Given a sequence of N CTCF binding sites, the number of patterns of length K is N-K+1. So, for example, if we have 10 binding sites and we are searching for 2-plets, we are going to have 10 - 2 + 1 = 9 bi-plet patterns. On the other hand, we will have 8 and 7 tri-plets and tetra-plets, respectively.*

*In our genome-wide calculation we divide the genome in 42 sequences of CTCF sites using the chromosome arms and their centromeres as division points. This means that, having 61,079 single CTCF sites (1-plets), will lead to 61079 + (¬2 + 1)*42 = 61037 2-plets, 61079 + (-3 + 1)*42 = 60995 3-plets; and 61079 + (-4 + 1)*42 = 60953 4-plets. These marginal counts are now also reported in Figure 2.*

*In the case of bi-plets, each pattern overlaps the adjacent one by one binding site, which means that some sequences of patterns are mandatory. A convergent pattern (><) can only be followed by a left-pointing same pattern (<<) or a divergent one (<>); and a divergent pattern (<>) can be followed only by a right-pointing same (>>) or a convergent bi-plet (><). This balancing imposes that in a linear sequence of CTCF sites, the number of divergent and convergent bi-plet patterns can differ by a maximum count of 1 or be zero. If we extend this possible skew to the entire set of 42 sequences, we can have a maximum skew of 42 patterns genome-wide (if only one of the two classes is consistently higher than the other). What happens in practice is that this 1-count skew is balanced between convergent and divergent patterns across all chromosome arm sequences, and in our case we end up with a global 1-count skew.*

*In the case of 3-plets, each pattern overlaps the adjacent one by two binding sites. Applying the same reasoning as for 2-plet patterns, it is easy to see that divergent and convergent 3-plets can differ by maximum 2 counts in a single sequence. These possible skews cancel each other across all 42 sequences and we end up also in this case with a global 1-count skew.*

*In the case of 4-plets, each pattern overlaps the adjacent one by three binding sites. Same logic applies, but in this case the observed global skew is of 80 counts.*

*An abridged version of this extended discussion of the numbers has been added to the methods of the manuscript.*

R2.3 In the Table of Fig.2, Mention « patterns » in the table on Top of the column containing « Same, Convergent, Divergent, Conv-Div ». This would avoid confusion in the text with « the number of possible permutations ».

*We amended Figure 2 as requested. See also previous point.*

R2.4 When the authors mention an evolutionary gain of same « oriented triplets », is there a way to measure this statistically when compared with convergent, Divergent and Conv-Div.

*This point has been answered in response to the reviewer 1 comment 3. Essentially, the statistical significance for the depletion of Conv.Div CTCF triplets and gain of Same CTCF triplets in the human genome vanishes to zero in the empirical CTCF orientation permutation tests displayed on Rebuttal Figure 11.*

R2.5 Fig.3B: randomization is performed, indicating that the « real » distribution is biased. The authors should calculate a statistical score associated with this analysis. Same remark for Figure 3C.

*In the original manuscript, for Figure 3C we performed the t-test of two of the pairwise correlations, which were reported in the main text “... convergent patterns tend to be larger than Same-oriented patterns (Pval = 1.12e-17) while divergent patterns tend to be smaller (Pval = 3.13e-13)...”. Following the suggestion of the reviewer, we have now added all the p-values for the comparisons with the Same pattern in the revised Manuscript Figure 3C version (Rebuttal Figure 18). P-values have been corrected with Bonferroni correction for multiple testing.*

*Rebuttal Figure 18: Updated Manuscript Figure 3 panel C, now including p-values for all the comparisons to the Same patterns and the comparison between divergent and convergent (see also rebuttal Figures 11-17).*

*In Figure 3B we are counting the number of clusters formed at each clustering window. While it is easy to calculate significance for the pattern class configurations (which we did in this rebuttal, answering Reviewer 1 question 3, Pearson chi-square test), building a random model of spatial clustering requires performing spatial randomization of the binding sites and that is a very intricate enterprise, if not hard. As an alternative, we validated the statistical significance of the difference between random and real spatial distribution of CTCF sites by analyzing pairwise distances between adjacent binding sites along the genome. This can be tested using a Mann–Whitney U test, checking if the real and random distributions are significantly different. As shown on Rebuttal Figure 19, the test is significant, with a p-value vanishing to zero. The real binding sites being more clustered together at small distances than expected. This is in concordance with recent studies about tandem CTCF binding sites and their mutual influence [2].*

*Rebuttal Figure 19: Observed density of CTCF sites as a function of genomic distance compared to a random distribution of 61,079 sites along the length of human chromosomes. Note that the orange curve is identical to the observed distribution plotted in the original Figure 3A of the submitted manuscript, which Rebuttal Figure 19 now replaces 3A in the revised manuscript.*

*One could argue that Figure 3B is indeed a discretization of the cumulative distribution of the distributions shown in the density plot above, where we compute the probability for a cluster to be formed when two binding sites have a distance less or equal to a window w. We have now substituted the original Figure 3A with Rebuttal Figure 19 and kept Figure 3B as before, in order to give the reader both the pairwise and cumulative distributions together with the Mann-Withney statistical validation as an inset in figure 3A.*

R2.6 line 184: the authors claimed that "Convergent di-plets are depleted when using the entire CTCF set (Fig.3E), but become enriched when the top quartile CTCF set is considered (Fig S2C)". However, this doesn't seem to be the case or may require some clarification.

*Reviewer 2 is correct and we are thankful for having spotted this confused statement. Accordingly, we deleted the text.*

R2.7 line 244: I was not sure how the authors calculate that 16% of S1-S7 boundaries lack a CTCF site. It is actually an important point to mention. Actually, it would have been nice to have the percentage for each « S » category in order to appreciate that the highly conserved boundaries are the most enriched in CTCF binding sites.

*The computation was done simply by intersecting boundaries with CTCF sites. We agree that the number is important, as not all inferred boundaries can be explained by CTCF sites. As shown on Figure 5A, there are fewer boundaries without CTCF sites within 25 kb of their center as boundary conservation increases. In the revised manuscript we now quote the percentages of boundaries without CTCF sites for the boundaries that are observed once and seven times (S1, S7).*

R2.8 Concerning epigenetic marks at TAD boundaries, the authors report a « weak but discernible enrichment of H3K27ac and H3K4me1 marks around some negative DI inversion sites ». Those marks often correspond to enhancers. Actually, a clearer enrichment of H3K4me3, the mark of TSS/promoters is also observed at this negative inversion sites inside TADs, which would indicate that gene promoters and enhancers could have a biased position inside TADs. Something to clarify/discuss in the text.

*The reviewer is correct. We elected to delete the sentence ‘Furthermore, there appears to be a weak but discernible enrichment of H3K27ac and H3K4me1 marks around some negative DI inversion sites (Fig. S3D).’ as the signals are very faint in this plot. Of course, there exist gene (features) that are at the negative DI sign inversion sites in ‘the middle of TADs’. However, this is neither a rule nor an exception.*

R2.9 For the discussion, their representation of TAD as <>(>...>)(<...<)<> with divergent CTCF sites at TAD boundaries and convergent CTCF sites inside TAD halves is clearly a novelty for the field.

*We are glad that Reviewer 2 acknowledges the novelty of our conclusions.*

R2.10 However, it is a pity that the authors don't take advantage of this finding to extend a bit their discussion toward an evolutionary perspective (see for example, Vietri Rudan et al, Cell Rep 2015 paper to extend the discussion part).

*We thank reviewer 2 for providing us with this opportunity to discuss our findings from an evolutionary perspective.*

*In order to put our CTCF nomenclature and in particular the results displayed on Figures 3 and supplementary figure 4 in the revised manuscript, we now state at the end of the discussion that; “The extreme statistical significance of the spatial imbalances between the spatial distributions of convergent and divergent CTCF configurations are robust even to removal of the 25% strongest CTCF sites. This suggests that there is evolutionary pressure to maintain the relative CTCF orientations that form the grammatical rules we discovered, in keeping with the conclusions drawn from CTCF site orientation conservation in syntenic regions of mouse and man [7].*

*References*

*1.	Cresswell KG, Dozmorov MG. TADCompare: An R Package for Differential and Temporal Analysis of Topologically Associated Domains. Front Genet. Frontiers Media S.A.; 2020;11:158.*

*2.	Jia Z, Li J, Ge X, Wu Y, Guo Y, Wu Q. Tandem CTCF sites function as insulators to balance spatial chromatin contacts and topological enhancer-promoter selection. Genome Biol [Internet]. BioMed Central Ltd.; 2020 [cited 2020 May 6];21:75. Available from: https://genomebiology.biomedcentral.com/articles/10.1186/s13059-020-01984-7*

*3.	Rao SSP, Huntley MH, Durand NC, Stamenova EK, Bochkov ID, Robinson JT, et al. A 3D map of the human genome at kilobase resolution reveals principles of chromatin looping. Cell [Internet]. Cell Press; 2014 [cited 2018 Nov 28];159:1665–80. Available from: http://www.ncbi.nlm.nih.gov/pubmed/25497547%0Ahttp://www.pubmedcentral.nih.gov/articlerender.fcgi?art id=PMC5635824*

*4.	Crane E, Bian Q, McCord RP, Lajoie BR, Wheeler BS, Ralston EJ, et al. Condensin-driven remodelling of X chromosome topology during dosage compensation. Nature. 2015;*

*5.	Abdennur N, Mirny LA. Cooler: scalable storage for Hi-C data and other genomically labeled arrays. Bioinformatics. 2019;*

*6.	Hashimoto H, Wang D, Horton JR, Zhang X, Corces VG, Cheng X. Structural Basis for the Versatile and Methylation-Dependent Binding of CTCF to DNA. Mol Cell. Cell Press; 2017;66:711-720.e3.*

*7.	Vietri Rudan M, Barrington C, Henderson S, Ernst C, Odom DT, Tanay A, et al. Comparative Hi-C Reveals that CTCF Underlies Evolution of Chromosomal Domain Architecture. Cell Rep. 2015;*

**Second round of review**

**Reviewer 1**

The authors have greatly tightened and improved the clarity of the manuscript, and I am now fully convinced of both the novelty and potential impact of the work. I support the manuscript's publication, but have two minor quibbles that I would still like to be addressed.

1. In the rebuttal to my previous point 2, the authors have convincingly demonstrated that promoter-linked CTCF sites are not necessarily the strongest, and so going from promoter-centered to genome-wide analyses does not drastically affect their conclusions. They also convincingly showed that DNA methylation is not really a confounding factor that needs to be considered in their analyses. But where I am still confused is why the aggregate ranking is even necessary, beyond showing that promoter-proximal CTCF sites do not require strong motifs to bind CTCF, presumably due to more open chromatin. That message is nice enough in itself, but somewhat detracts from the main point about CTCF grammar in organizing chromosome architecture. Does this motif/occupancy decoupling alter any further assumptions of loop extrusion models? I would naively expect CTCF occupancy (via ChIP-seq data) to be sufficient, since CTCF presence is required to impede extrusion, regardless of the strength of the underlying motif. Or am I missing something?

2. Even though it is "negative" data, the result that DNA methylation does not confound the analysis should be put in the manuscript somehow as a supplementary figure. If my understanding of the authors' analysis is right, this analysis is currently quite biased to the promoters (based on available data), and since CTCF binding has been demonstrated to be affected by DNA methylation, people in the community may naively think that TADs could be engineered by targeted DNA methylation/demethylation. The supplemental figure, while "negative", could be informative here.
